# Complementarity of empirical and process-based approaches to modelling mosquito population dynamics with *Aedes albopictus* as an example—Application to the development of an operational mapping tool of vector populations

**DOI:** 10.1371/journal.pone.0227407

**Published:** 2020-01-17

**Authors:** Annelise Tran, Morgan Mangeas, Marie Demarchi, Emmanuel Roux, Pascal Degenne, Marion Haramboure, Gilbert Le Goff, David Damiens, Louis-Clément Gouagna, Vincent Herbreteau, Jean-Sébastien Dehecq

**Affiliations:** 1 CIRAD, UMR TETIS, Sainte-Clotilde, Reunion, France; 2 TETIS, Univ Montpellier, AgroParisTech, CIRAD, CNRS, INRAE, Montpellier, France; 3 CIRAD, UMR ASTRE, Sainte-Clotilde, Reunion, France; 4 ASTRE, Univ Montpellier, CIRAD, INRAE, Montpellier, France; 5 IRD, UMR ESPACE-DEV, Montpellier, France; 6 Maison de la Télédétection, Montpellier, France; 7 UMR MIVEGEC, IRD, Sainte-Clotilde, Reunion, France; 8 Regional Health Agency, Sainte-Clotilde, Reunion, France; INRA, FRANCE

## Abstract

Mosquitoes are responsible for the transmission of major pathogens worldwide. Modelling their population dynamics and mapping their distribution can contribute effectively to disease surveillance and control systems. Two main approaches are classically used to understand and predict mosquito abundance in space and time, namely empirical (or statistical) and process-based models. In this work, we used both approaches to model the population dynamics in Reunion Island of the 'Tiger mosquito', *Aedes albopictus*, a vector of dengue and chikungunya viruses, using rainfall and temperature data. We aimed to *i)* evaluate and compare the two types of models, and *ii)* develop an operational tool that could be used by public health authorities and vector control services. Our results showed that *Ae*. *albopictus* dynamics in Reunion Island are driven by both rainfall and temperature with a non-linear relationship. The predictions of the two approaches were consistent with the observed abundances of *Ae*. *albopictus* aquatic stages. An operational tool with a user-friendly interface was developed, allowing the creation of maps of *Ae*. *albopictus* densities over the whole territory using meteorological data collected from a network of weather stations. It is now routinely used by the services in charge of vector control in Reunion Island.

## Introduction

Mosquito-borne diseases result from pathogens transmitted to humans or animals by mosquito bites. They place heavy health and economic burdens on the countries where they are present [1]. Moreover, over recent decades mosquito-borne diseases such as dengue, chikungunya, and Zika, all caused by *Aedes* species, have emerged or re-emerged in many regions [2–4]. As vaccines do not exist for most mosquito-borne diseases, vector control is essential to prevent outbreaks. A capacity to predict the locations and dynamics of mosquito populations at a local scale would help vector control agencies target their interventions.

Modelling approaches are powerful tools for identifying and prioritizing where and when surveillance and control should be targeted. Two main approaches are used to understand and predict mosquito population dynamics: *i)* process-based (or mechanistic) models describing biological knowledge within a mathematical or computational framework, and *ii)* empirical (or statistical) models, which try to find, from the observed data, a predictive function of the response variable (mosquito populations) based on a set of predictors within a statistical or a machine learning framework. Both approaches have been successfully applied to different mosquito species and geographical contexts [5–17], resulting in a better understanding of their distribution [5–8, 11, 12, 16] and dynamics [9, 10, 13, 17, 18] and the assessment of different mosquito control strategies [19, 20]. However, most case studies only develop one of the two approaches (either empirical [5–8, 11, 12, 14, 16] or process-based [9, 10, 13, 15, 17] depending on the availability of data and knowledge), and do not compare the capacity of the two approaches to predict mosquito population dynamics. Moreover, although many models have been developed, including spatially explicit simulation models (*e*.*g*., ‘Skeeter buster’ software for *Aedes aegypti* [15]), there is a lack of operational tools that can be used by public health authorities and vector control services.

In Reunion island, a French overseas department located in the Indian Ocean, *Aedes (Stegomyia) albopictus* (Skuse) (Diptera: Culicidae) caused two major arbovirus outbreaks in 1977 (dengue) and in 2005–2006 (chikungunya) [3, 21]. In 2004, a second dengue outbreak of lower intensity was reported, followed by an interepidemic period with sporadic cases and clusters [22]. Recently, a recrudescence of dengue occurred, with 7596 new autochthonous dengue cases reported between 2017 and February 2019 [23]. This epidemic situation heightened the needs of the vector control service of the Regional Health Agency (local representation of the French Ministry of Health) for predictive spatial models of *Ae*. *albopictus* population dynamics. Indeed, very high spatial and temporal heterogeneities of *Ae*. *albopictus* density have been reported [24]. Different modelling studies of *Ae*. *albopictus* distribution and dynamics have been developed in recent years. Distribution maps have been derived from environmental and meteorological datasets using empirical models, at worldwide [7], regional [8], national [11, 12] and local scales [6, 16]. In Reunion Island, theoretical process-based population dynamics models have been used to assess control strategies [20, 25], taking into account mosquito dispersal [26, 27] and the impact of human behavior [28]. Nevertheless, none of these precedent research efforts have addressed both space and time dynamics of *Ae*. *albopictus* to produce mosquito density maps that can be used by the public health authority to target vector surveillance and control actions.

Our objective was twofold. First, we developed and compared two types of models of *Ae*. *albopictus* species population dynamics in Reunion Island: an empirical model that predicts mosquito population dynamics from a set of weather variables using a Support Vector Machine (SVM) [29], and a process-based model derived from (Cailly et al., 2012; Tran et al., 2013) [17, 19]. The comparison was performed by confronting the predictions of the two models against field entomological data. Second, from the results of the two modelling approaches, we developed, in close collaboration with the vector control service of the Regional Health Agency (https://www.ocean-indien.ars.sante.fr/), an operational tool that produces maps of predicted *Ae*. *albopictus* abundances from daily rainfall and temperature data collected from the National Weather Service (https://donneespubliques.meteofrance.fr/).

## Materials and methods

### Study area

**Fig 1:**
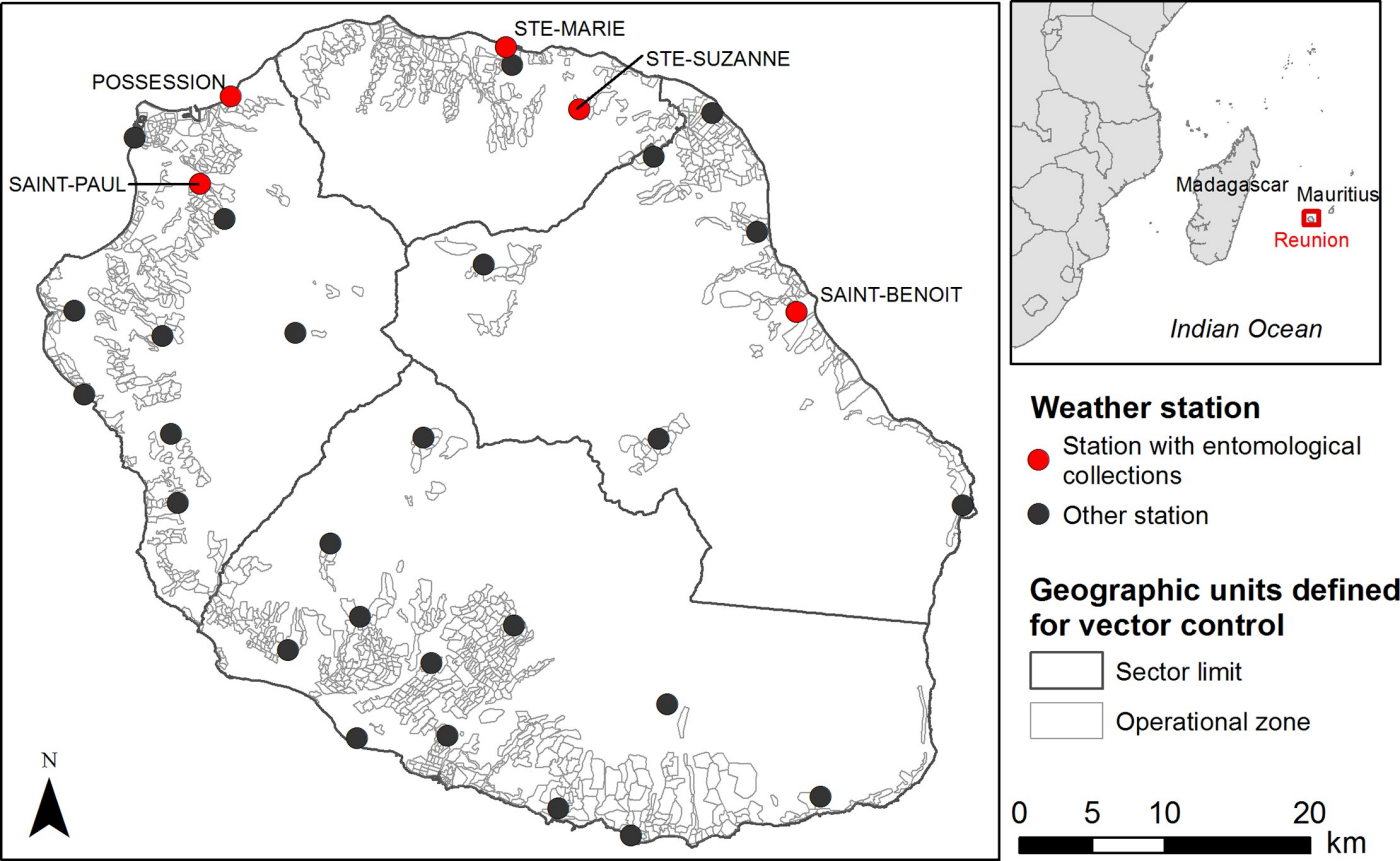
Location of the study area, Reunion Island.

Reunion Island (2,500 km^2^, 865,826 inhabitants in 2018) is a French overseas department located in the Indian Ocean, 700 km east of Madagascar and 175 km southwest of Mauritius (Fig 1). The climate is tropical with hot and rainy summers (November–April) and warm and dry winters (May–October). With a peak at 3,070 meters and a rugged relief, rainfall and temperatures vary greatly depending on the hillsides and altitudes: the eastern side of Reunion has very high rainfall while the western side is drier. The temperature decreases progressively from the coast to the central mountains. Our study area was delimited by the 1,203 operational zones defined by the vector control service of the Regional Health Agency, corresponding to inhabited areas where surveillance and control actions are organized into four operational sectors (East, West, North and South) (Fig 1). These operational zones cover all of the populated areas of the island where *Ae*. *albopictus* is present and a threat for public health. They were defined to include about 200 households (the average is 238 households per operational zone), with a mean area of 33 ha (minimum = 3 ha; maximum = 340 ha) (S1 Fig).

### Entomological data

Larval and pupae collections were performed weekly between February and June 2012 (two sites: Ste-Marie and St-Benoit) and November 2012 to March 2013 (four sites: Ste-Marie, Ste-Suzanne, Possession, St-Paul) (Fig 1, Table 1). The municipalities issued the permission to the regional Health Agency, representative of the French Ministry of Health, to conduct the field studies in the framework of its public health mission. In each site, six traps (small containers of 100 cl) were deployed in the vicinity of a national weather station. We considered that each study site covered a 300 m-radius area around the corresponding weather station (28.3 ha), a distance corresponding to the active dispersal flight of *Ae*. *albopictus* [30–35]. The traps were placed empty at the beginning of the trapping campaign, and then were naturally filled by rainfall. Each week, the number of larvae (L3-L4 stages) and pupae were recorded in the field (S1 Table). After being counted, the larvae and pupae were removed from the traps. In addition, egg collections were used for the validation of the process-based model (S1 File).

**Table 1:** Geographic coordinates of study sites.

Site	Latitude	Longitude
**La Possession**	-20.921	55.346
**St-Benoit**	-21.058	55.719
**St-Paul**	-20.975	55.325
**Ste-Marie**	-20.892	55.528
**Ste-Suzanne**	-20.931	55.576

### Meteorological data

The meteorological service ‘Meteo France’ (https://publitheque.meteo.fr) provided the daily temperature (minimum and maximum) and rainfall records from 2011 to 2014 at 32 weather stations, including the five weather stations where entomological data were collected (Fig 1). Indeed, although entomological data were collected between 2012 and 2014 (see above and S1 File), the acquisition of meteorological data covering the year before the period of interest was necessary for both models. The data was used for the calculation of climatic variables to be tested in the empirical approach, and for the initialization of the process-based model.

### Empirical approach

For all sites and all dates of entomological collections, the mean number of larvae (at the L3 and L4 stages) per trap was calculated. The purpose of the model was to predict these observations according to weather conditions. The weather conditions were featured by a combination of at most three input variables in order to avoid overfitting problems. The three input variables were drawn from a set of 531 climate indices based on minimum temperature, maximum temperature and rainfall, identified in the literature as potentially influencing *Ae*. *albopictus* abundance. These indices are:

- minimum temperature (last *N* days)
- maximum temperature (last *N* days)
- rain accumulation (last *N* days)
- maximum rainfall (last *N* days)
- maximum number of consecutive days without rain (last *N* days)
- number of days when variable *X* is greater or less than *S*_*X*_ (last *N* days)
- maximum number of consecutive days when variable *X* is greater or less than *S*_*X*_ (last *N* days)

with the retroactive period (last *N* days) that starts at the capture date and where *N* ∈ *[7*,*14*,*21*,*…63 days]*, *X* denoting either rainfall accumulation, minimum temperature, maximum temperature and *S*_*X*_ ∈ *[10*,*20*,*…90 percentile of X]*.

The model with the best performance was identified using a selection criterion based on the mean square error associated with a k-fold cross validation technique with k = 5. A Support Vector Machine (SVM) [36] is a supervised learning technique that analyses data and identifies patterns used for classification or regression. SVM maps input vectors (here the weather variables) to a higher dimension feature space using kernel functions in order to find maximum separating hyperplanes. Here we used the SVM version called Support Vector Regression (SVR) able to perform regression with continuous variables (here the mean number of larvae per trap). The SVR method was used to perform the non-linear regression with a radial kernel, a cost coefficient set to 1 and the epsilon parameter set to 1. All of the statistical calculations were performed in R language [37] using the package “e1071” [29]. For some input variables such as the ones related to rain accumulation, a logarithm was applied to obtain a Gaussian-like distribution which gave better results in terms of mean square error.

### Process-based approach

**Fig 2:**
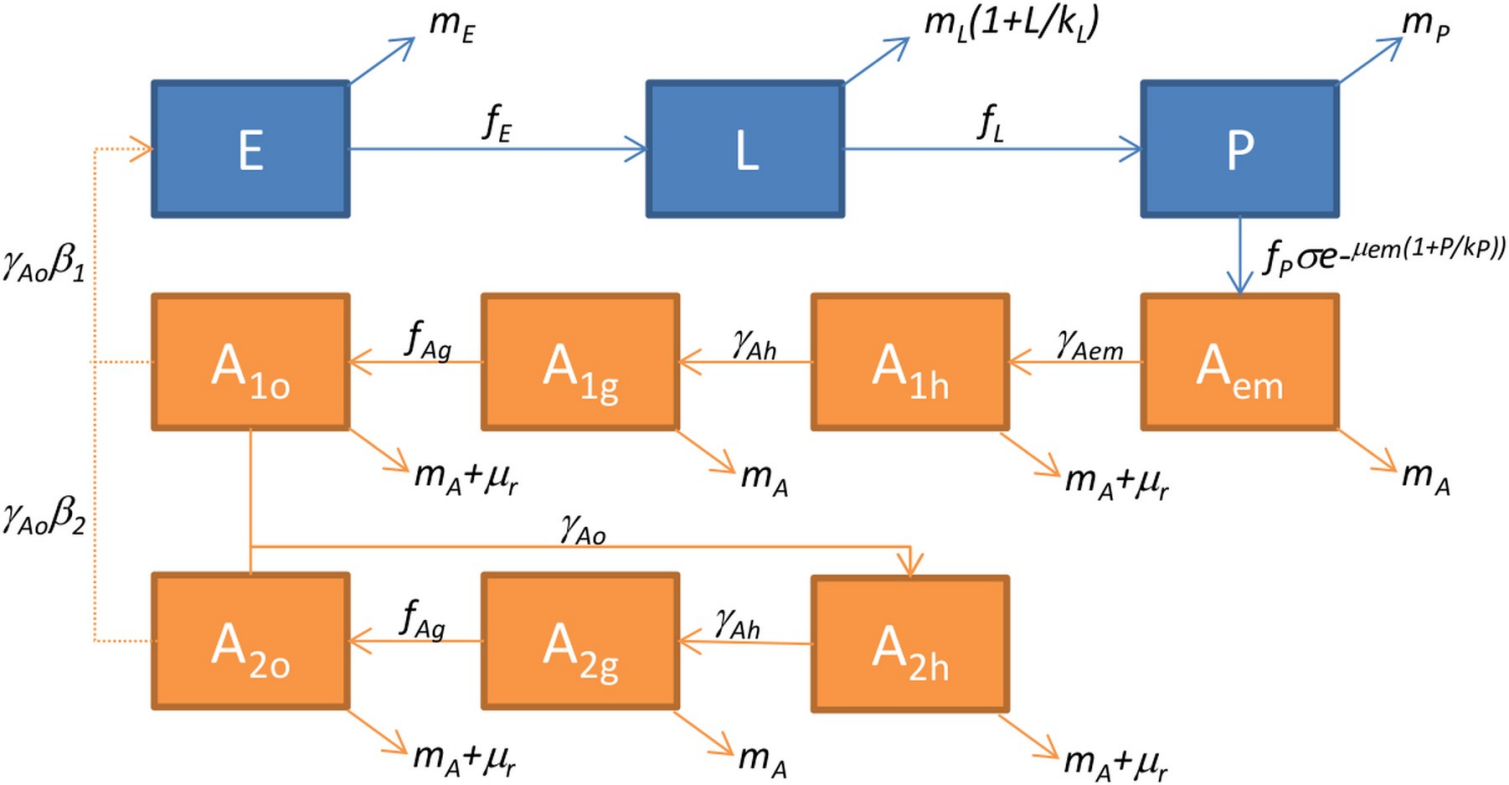
Diagram of the process-based model of *Aedes albopictus* population dynamics. In blue, the aquatic stages (*E*: eggs, *L*: larvae, *P*: pupae); in orange, the adult female stages (*A*_*em*_: emerging, *A*_*1*_: nulliparous, *A*_*2*_: parous, with *h*: host-seeking, *g*: resting, *o*: ovipositing).

We used the generic mechanistic framework proposed by Cailly et al. [10, 19] for modelling mosquito populations that was adapted to *Ae*. *albopictus* in southern France [17]. We followed the recommendations of (Ezanno et al., 2015) [10] to adapt the generic population dynamics model in a new geographic area. The model is based on a system of ordinary differential equations (ODE) and represents all steps of the mosquito life cycle, considering both aquatic stages (*E*, eggs; *L*, larvae; *P*, pupae) and adult stages (*A*_*em*_, emerging adult females; *A*_*1*_, nulliparous females; *A*_*2*_, parous females). In addition, parous and nulliparous females are subdivided in compartments regarding their behaviour (h, host-seeking; g: transition from engorged to gravid; o, oviposition site seeking) (Fig 2).

The mosquito life cycle used in [17] was modified because the tropical strains of *Ae*. *albopictus* that are present in Reunion Island do not enter diapause and are active throughout the year [38]. Thus, the ODE system is:

$$\left\{ \begin{array}{c} \dot{E}=\gamma_{Ao}(\beta_{1}A_{1o}+\beta_{2}A_{2o})-(m_{E}+f_{E})E\\ \dot{L}=f_{E}E-(m_{L}(1+L/k_{L})+f_{L})L\\ \dot{P}=f_{L}L-(m_{P}+f_{P})P\\ \dot{A_{em}}=f_{P}P\sigma e^{\left(-\mu_{em}(1+P/k_{P})\right)}-(m_{A}+\gamma_{Aem})A_{em}\\ \dot{A_{1h}}=\gamma_{Aem}A_{em}-(m_{A}+\mu_{r}+\gamma_{Ah})A_{1h}\\ \dot{A_{1g}}=\gamma_{Ah}A_{1h}-(m_{A}+f_{Ag})A_{1g}\\ \dot{A_{1o}}=f_{Ag}A_{1g}-(m_{A}+\mu_{r}+f_{Ao})A_{1o}\\ \dot{A_{2h}}=f_{Ao}(A_{1o}+A_{2o})-(m_{A}+\mu_{r}+\gamma_{Ah})A_{2h}\\ \dot{A_{2g}}=\gamma_{Ah}A_{2h}-(m_{A}+f_{Ag})A_{2g}\\ \dot{A_{2o}}=f_{Ag}A_{2g}-(m_{A}+\mu_{r}+\gamma_{Ao})A_{2o} \end{array}\right.$$ (1)

Parameters and functions were adapted from [17] given the results of experimental [39] and observational [24, 40] studies on local *Ae*. *albopictus* populations in Reunion. Parameters (Greek letters in Eq 1) are constant: for stage X, *γ*_*X*_ is the transition rate to the next compartment, *μ*_*X*_ the mortality rate, *β*_*X*_ the egg laying rate and *σ* the sex-ratio at the emergence; *μ*_*r*_ is an additional adult mortality rate related to seeking behaviour (Table 2). According to the sensitivity analysis of the model (see [17] for details), the standard environment carrying capacity, the mortality rate at emergence, and the sex-ratio, are the most influential parameters on the variations in the peak of adult abundance, and need to be estimated as precisely as possible.

**Table 2:** Process-based approach: model parameters.

Notation	Definition	Value	Reference
*β*_1_	Number of eggs laid/ovipositing nulliparous female	60	[39]
*β*_2_	Number of eggs laid/ovipositing parous female	80	[39]
*σ*	Sex-ratio at emergence	0.5	[39]
*γ*_*Aem*_	Development rate of emerging adults (day^−1^)	0.4	[17]
*γ*_*Ah*_	Transition rate from host-seeking to engorged adults (day^−1^)	0.2	[17]
*γ*_*Ao*_	Minimum transition rate from ovipositing to host-seeking adults (day^−1^)	0.2	[17]
*μ*_*E*_	Minimum egg mortality rate (day^−1^)	0.05	[17]
*μ*_*em*_	Mortality rate during emergence (day^−1^)	0.1	[17]
*μ*_*r*_	Mortality rate related to seeking behaviour (day^−1^)	0.08	[17]
*T*_*E*_	Minimal temperature needed for egg development (°C)	10	[17]
*TDD*_*E*_	Total number of degree-day necessary for egg development (°C)	110	[17]
*T*_*Ag*_	Minimal temperature needed for egg maturation in females (°C)	10	[17]
*TDD*_*Ag*_	Total number of degree-day necessary for egg maturation (°C)	77	[17]
*κ*_*Lfix*_	Standard rainfall-independent environment carrying capacity for larvae	Field observations	
*κ*_*Lvar*_	Standard rainfall-dependent environment carrying capacity for larvae	Field observations	
*κ*_*Pfix*_	Standard rainfall-independent environment carrying capacity for pupae	Field observations	
*κ*_*Pvar*_	Standard rainfall-dependent environment carrying capacity for pupae	Field observations	

Functions (Latin letters in Eq 1) are weather-driven functions, varying over time: for stage *X*, *f*_*X*_ is the transition rate to the next stage, *m*_*X*_ the mortality rate, *k*_*X*_ the environment carrying capacity. Consistent with the empirical approach, we considered temperature (*T*) and precipitation (*P*) as the two forcing function variables (Table 3). Daily precipitation and daily mean temperature were used. Temperatures affect the development of aquatic stages, egg maturation, and the mortality rates of larvae, pupae and adults [39]. Heavy rains have an impact on the mortality rates of aquatic stages by flushing the breeding habitats [41]. Rainfall also has an impact on the availability of breeding sites in the environment, and thus the transition rate from ovipositing to host-seeking female adults [17, 42].

**Table 3:** Process-based approach: model functions.

Notation	Definition	Expression	Reference
*f*_*E*_	Transition function from egg to larva	$\left\{ \begin{array}{cc} (T(t)-T_{E})/{TDD}_{E} & if\;T(t)>T_{E}\\ 0 & otherwise \end{array}\right.$	[17]
*f*_*L*_	Transition function from larva to pupa	*q*_1_*T*^2^+*q*_2_*T*+*q*_3_ with q1 = -0.0007; q2 = 0.0392; q3 = -0.3911	[17]
*f*_*P*_	Transition function from pupa to emerging adult	*q*_1_*T*^2^+*q*_2_*T*+*q*_3_ with q1 = -0.0008; q2 = -0.0051; q3 = -0.0319	[17]
*f*_*Ag*_	Transition function from engorged adult to oviposition site-seeking adult	$\frac{T(t)-T_{Ag}}{{TDD}_{Ag}}$	[17]
*f*_*Ao*_	Transition function from ovipositing to host-seeking adults (day^−1^)	*γ*_Ao_*(1+*P*_*norm*_)	[42]
*m*_*E*_	Egg mortality	$\mu_{E}+\left\{ \begin{array}{cc} 0.1 & if\;P>80\\ 0 & otherwise \end{array}\right.$	[17, 41]
*m*_*L*_	Larva mortality	${0.02+0.0007\;e}^{0.1838(T-10)}+\left\{ \begin{array}{cc} 0.5 & if\;P>80\\ 0 & otherwise \end{array}\right.$	[39, 41]
*m*_*P*_	Pupa mortality	${0.02+0.0003\;e}^{0.2228(T-10)}+\left\{ \begin{array}{cc} 0.5 & if\;P>80\\ 0 & otherwise \end{array}\right.$	[39, 41]
*m*_*A*_	Adult mortality	0.025+0.0003*e*^0.1745(*T*−10)^	[39]
*k*_*L*_	Environment carrying capacity for larvae	Eq 2	[17]
*k*_*P*_	Environment carrying capacity for pupae	Eq 2	[17]

In Reunion Island, *Ae*. *albopictus* can be found in urban, suburban and natural areas up to an altitude of 1,200 meters [40]. Females lay eggs in either natural (*e*.*g*., tree holes, bamboo stumps) or artificial (*e*.*g*., water containers, flower plates or vases, basins and reservoirs, tires) oviposition sites [24]. Thus, the environment carrying capacity of aquatic stages (*k*_*L*_, *k*_*P*_) that reflects the availability of oviposition sites in a given place is partially driven by precipitation: due to artificial flooding (*e*.*g*., watering of gardens), human-made oviposition sites such as flower plates or vases provide a standard environment carrying capacity for larvae or pupae (*κ*_*Xfix*_) that remains constant over time, whereas the availability of the other types of oviposition sites is driven by rainfall (Eq 2):

$$k_{X}(t)=\kappa_{Xfix}+\kappa_{Xvar}*P_{norm}(t),X\;in\;\{L;P\}$$ (2)

The standard fixed (*κ*_*Xfix*_) and variable (*κ*_*Xvar*_) environment carrying capacities, which are key parameters of the model, were estimated for each Regional Health Agency operational zone from field observations that are routinely collected by the vector control service (see S2 File for details). *P*_*norm*_*(t)* is defined as the rainfall amount summed over a one week period, and normalized in order to vary between 0 and 1 [17].

### Evaluation of the models

The two models were evaluated by comparing the predicted abundance of larvae with the abundance observed through entomological data collections in the five study sites. For each modelling approach and each site, the number of trapped mosquitoes over time was compared to the model’s predictions for larvae using Spearman’s correlation coefficient. This analysis was relevant for comparing time-series. For the empirical model, the estimated number of larvae per trap was compared to the observed number of larvae per trap. For the process-based model, we compared relative abundances (*i*.*e*., the predicted larvae density against the observed number of larvae per trap). Indeed, absolute quantitative information on larvae abundance in the field is not available. The same method was used to compare egg abundances as predicted by the process-based model with egg collections (S1 and S3 Files).

To assess the agreement between the two models under different climatic conditions, empirical and process-based models’ predictions were compared using Spearman’s correlation coefficient from the data of the 32 weather stations (Fig 1).

### Development of the ‘ALBORUN’ tool

Spatial dynamic models of *Ae*. *albopictus* populations were built within the ‘Ocelet’ language and simulation environment (www.ocelet.org). This programming language is dedicated to the modelling of spatially explicit systems and their dynamics and facilitates the handling of spatial information using interaction graphs [43]. In the ‘ALBORUN’ model, the main elements of the models (‘entities’) are *i)* the 1,203 operational zones defined by the vector control service (polygon geometry), characterized by their respective values of standard fixed (*κ*_*Xfix*_) and variable (*κ*_*Xvar*_) environment carrying capacities, and *ii)* the 32 weather stations (point geometry) whose daily rainfall and temperature are imported as text file (csv format). Operational zone entities interact with the weather stations through a ‘relation’: the temperature and rainfall of each operational zone is defined as those of the closest weather station. In the ‘scenario’, the sequence of operations and interactions between operational zones and weather stations are defined as follows: *i)* the daily rainfall, and the minimum and maximum temperature values are read from the weather stations and attributed to the related operational zones; *ii)* for each operational zone, the model functions (Table 3) are updated; *iii)* the process-based *Ae*. *albopictus* population dynamics are computed (Eq 1) using the implicit Euler method to solve the ODE; *iv)* the significant variables identified by the empirical approach are updated; and *v)* the empirical approach-based predicted *Ae*. *albopictus* population dynamics are computed. The computation of predicted *Ae*. *albopictus* densities through the different approaches enables the user to compare the two models' outputs for the larval stage. The Ocelet source code is available on Github (https://github.com/OceletTeam/ocelet) and the ‘ALBORUN’ files are available in a dataverse repository (doi:10.18167/DVN1/XF2I3L).

To provide an operational tool for the staff of the vector control service of the Regional Health Agency, a user-friendly interface was developed using Xthe JavaFx library (https://docs.oracle.com/javafx). The Ocelet simulation environment automatically translates the model written in Ocelet language into a program written in Java programming language (www.java.com). That program is saved in the form of a java archive (jar) file which can be embedded into the JavaFx based user interface.

## Results

### Empirical model

**Fig 3:**
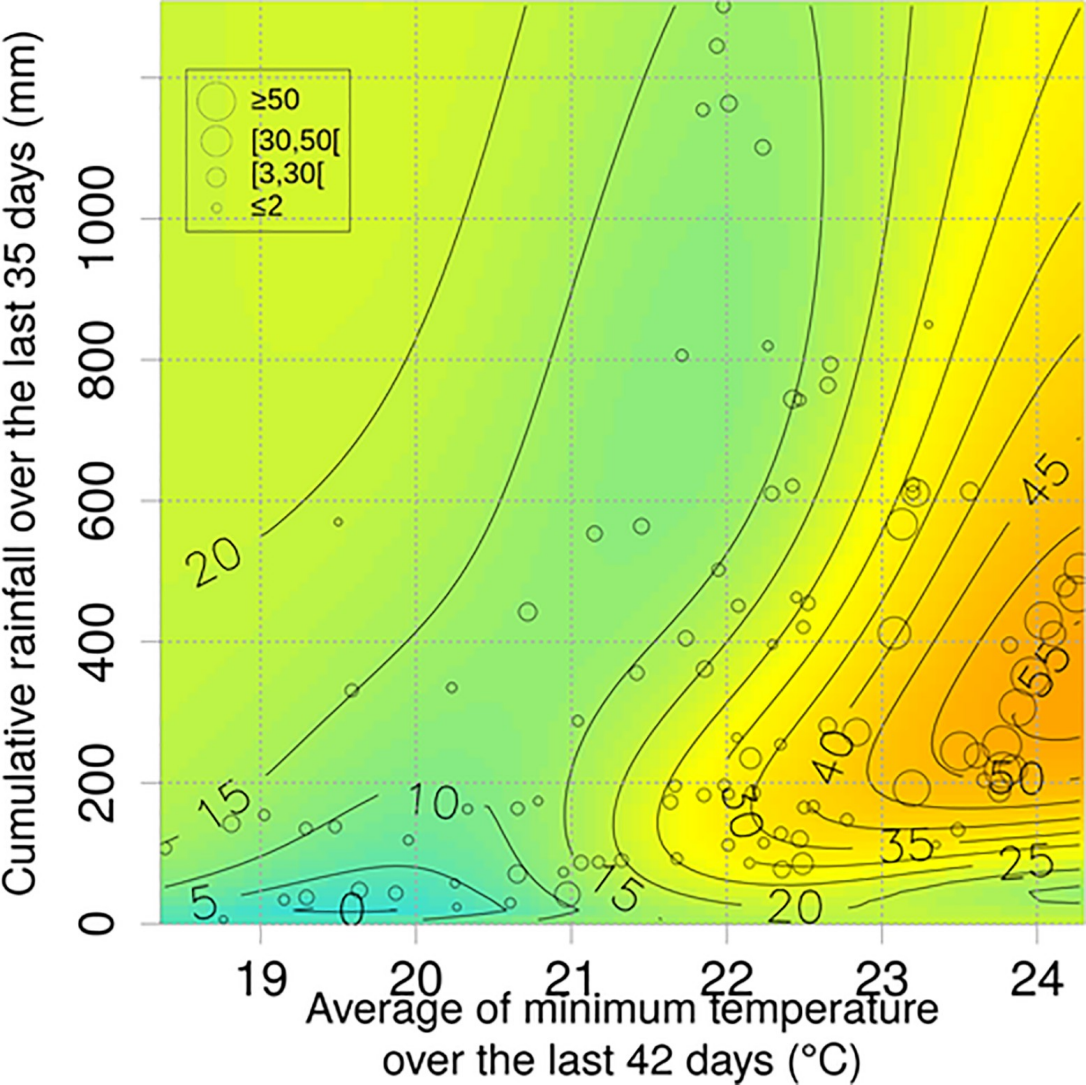
Prediction of the mean number of larval stages L3 and L4 per trap according to two variables: the cumulative rainfall over the last 35 days and the average of minimum temperature over the last 42 days. The colors and the level lines are related to the model predictions. The circles correspond to the observations. The size of the circles is proportional to the number of larvae observed considering the climatic conditions.

**Fig 4:**
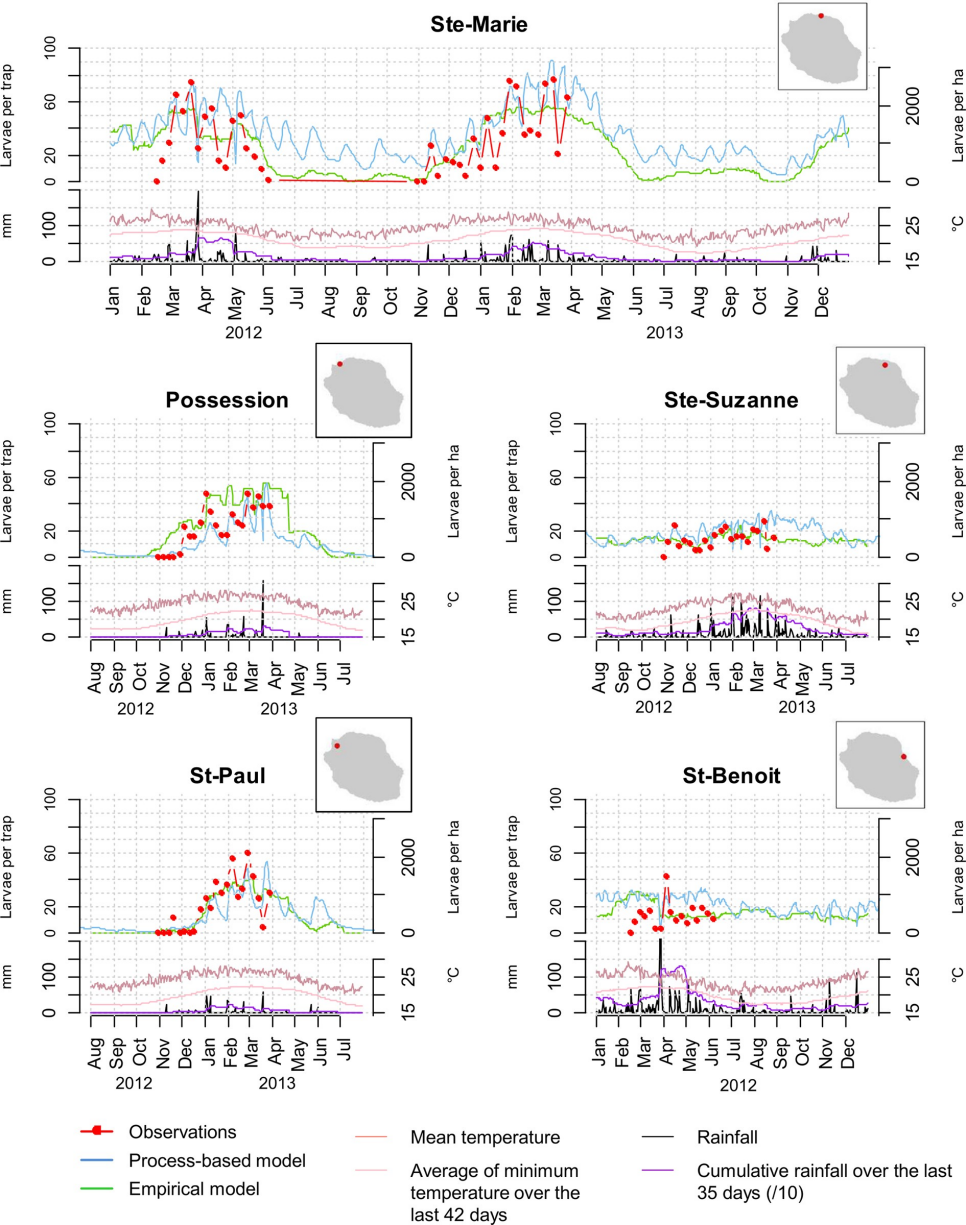
Comparison of observed and predicted abundances in *Aedes albopictus* larvae from rainfall and temperature data at different sites in Reunion Island, 2012–2013. The number of larvae per trap (L3 + L4 stages) and the larvae density (larvae per ha) are predicted by the empirical and process-based models, respectively.

The model with the best performance (S2 Table) included a set of two variables: the cumulative rainfall over the last 35 days and the average of minimum temperature over the last 42 days (Fig 3). The relationships between the juvenile population of *Ae*. *albopictus*, temperature and precipitation are clearly non-linear and the variations of mosquito abundances are very sensitive to those of temperature and precipitation. The favourable conditions are between 90 and 800 mm of rain accumulation over the last 35 days and an average of minimum temperature above 21°C over the last 42 days. Beyond 90 mm or over 800 mm of rainfall accumulation during the last 35 days, the number of L3 and L4 stage larvae decreases. The model fits seasonal variations in mosquito abundance, with a peak in March-April in late southern summer (Fig 4).

### Process-based model

Driven by daily rainfall and temperature data, the process-based model predicted the abundance of *Ae*. *albopictus* per stage (eggs, larvae, pupae, and nulliparous and parous female adult stages) over time. Different temperature and rainfall profiles resulted in variations of the mosquito population dynamics. The model predicted adequately the seasonal and interannual variations in the abundance of the aquatic stages, with a peak occurring in March, at the end of the austral summer, except for the St-Benoit site (Fig 4). Differences between sites were due to differences of rainfall profiles between the eastern and western sites, with heavier and regular rain events in the east. The maximum larvae densities were predicted for the northern site (Ste-Marie) (Fig 4).

### Evaluation of the models

The predictions of both the empirical and process-based approaches were consistent with the observed *Ae*. *albopictus* larvae abundance in the five collection sites (Fig 4). The predicted and observed abundances were highly correlated in the sites with higher larval densities and marked seasonal variations (St-Paul, Possession, Ste-Marie) (Table 4). In the eastern site (St-Benoit), where the observed *Ae*. *albopictus* abundances are low (<20 larvae/trap) with few seasonal variations, the correlation coefficients were lower and not significant. The predictions of the process-based model for egg dynamics were significantly correlated with observed egg abundances (see S3 File). The prediction accuracy of the empirical model deteriorated slightly when the 5 fold method is used or when the calibration is performed over the full dataset leaving out the data from the site in question. These results validated the robustness of the model when used on areas not included in the model calibration even if, as expected, the forecasts can be slightly less accurate in this case.

**Table 4:** Comparison of model predictions and entomological observed data at five sites in Reunion Island.

Site	Observed abundances [min–max]	Process-based model	Empirical model*			
		Spearman r (p-value)	Full dataset Spearman r (p-value)	Full dataset RMSE	5-fold dataset (random) RMSE	Leave one site out RMSE
St-Paul	[0–60.4]	0.82 (<10–5)	0.89 (<10^−5^)	12.0	13.2	16.7
Possession	[0–47.8]	0.89 (<10–5)	0.75 (<10^−4^)	9.6	10.6	14.8
Ste-Marie	[0–76.8]	0.62 (<10–4)	0.74 (<10^−5^)	19.1	19.7	22.3
Ste-Suzanne	[6.2–27.2]	0.65 (<10–4)	0.66 (10^−3^)	5.9	7.4	6.7
St-Benoit	[3–42.3]	0.45 (0.19)	0.33 (0.20)	11.8	12.9	15.5

* For the empirical model, Root Mean Square Errors are reported for each site when the model is calibrated using the full data set, a 5 fold method, and when the model is calibrated using all data except the site in consideration.

The comparison of the models’ predictions for 32 weather stations showed that the two models strongly agree for the areas with the highest mean annual temperatures (>22°C), corresponding to the western, northern and southern coastal areas, and can be in disagreement for the areas with low mean annual temperature (<19°C) or high annual rainfall (>4000 mm), corresponding to high elevation areas and the eastern coast (S2 and S3 Figs).

### ‘ALBORUN’ simulation tool

With a user-friendly interface (‘ALBORUN’ tool, see S4 File), the simulation model displays several input parameters that are used to control the simulations from the user interface. The users’ choices include the time interval and the geographical area (operational sectors) to run the simulations, as well as the input folders (the folders where input data are stored as shapefiles for weather stations and operational zones, and csv text files for daily weather data), and the output folder (the folder where outputs are saved as a shapefile for visualization with a Geographic Information System and a log file).

### Spatial model outputs

**Fig 5:**
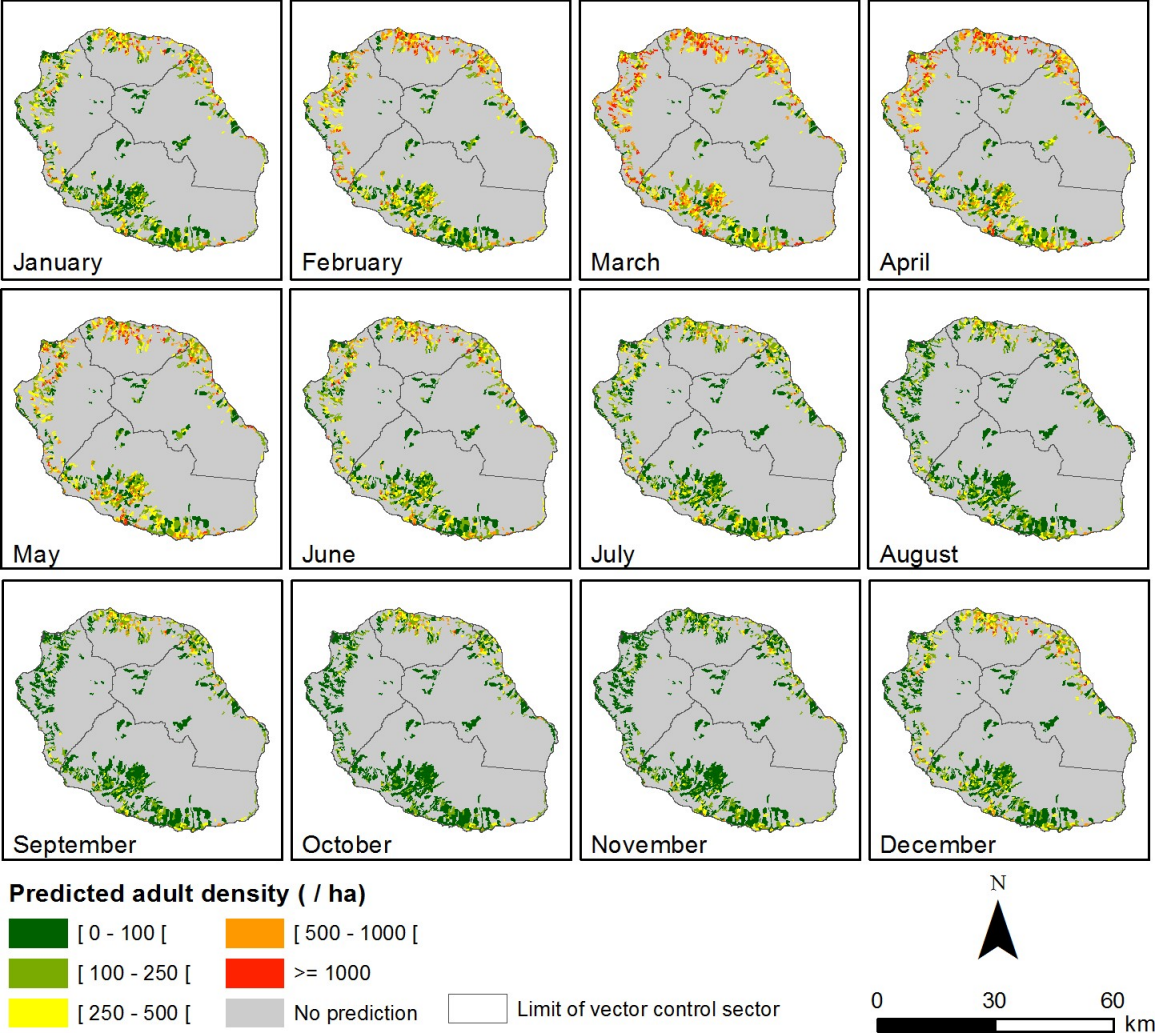
Regional maps of predicted *Ae*. *albopictus* abundances using ALBORUN tool (process-based model), Reunion Island, 2013.

Using daily precipitation and temperature data collected from a network of weather stations and the ‘ALBORUN’ tool, empirical and process-based models are used to simulate the spatial dynamics of *Ae*. *albopictus* population over Reunion Island, at a spatial scale adapted to vector control interventions (Fig 5). For each operational zone defined by the vector control services, the ‘ALBORUN’ tool predicts the abundances of *Ae*. *albopictus* mosquitoes per stage at a weekly frequency (this frequency was defined in consultation with the vector control service). The resulting maps highlight the high spatial and temporal heterogeneity of *Ae*. *albopictus* populations on Reunion Island (see example for year 2013 in Fig 5). Higher densities of *Ae*. *albopictus* are predicted during the austral summer, with a peak in March, when the species is present in high abundances in all coastal regions. ‘ALBORUN’ outputs (*Ae*. *albopictus* predicted abundances) in shapefile format can be integrated in a GIS environment with other geographical information (*e*.*g*. epidemiological data, administrative limits, records of previous vector-control operations, etc.) and used by public health stakeholders for visualization and analysis at different spatial scales (e.g. operational sector, municipality, or region).

## Discussion

The surveillance and control of mosquito-borne diseases is of prime public health importance in tropical areas. In Reunion Island, following the major chikungunya outbreak in 2005–2006 that had important health and economic impacts [44], and the recent dengue recrudescence [45, 46], there is an urgent need for operational mapping tools to optimize the actions of the vector control services. Our results demonstrate that modelling approaches may provide efficient and operational tools to depict vector population dynamics from environmental data in space and time.

### Environmental drivers of *Ae*. *albopictus* populations

Using only two input variables, rainfall and temperature, both the empirical and process-based modelling approaches successfully predicted *Ae*. *albopictus* in five contrasting locations of Reunion Island (Fig 4). These two variables have been identified as the main drivers of *Ae*. *albopictus* species in different geographic contexts [47–49]. According to the results of the empirical approach (Fig 3), higher temperatures favour larvae abundance. Indeed, temperature positively affects the development and survival of all aquatic stages up to 35° C [49]. This threshold value corresponds to the maximum temperature observed in Reunion Island (Fig 4), explaining the positive correlations between temperature and abundance of *Ae*. *albopictus* larvae in our study area. Our results also showed that precipitation impacts the abundance of *Ae*. *albopictus* mosquitoes in Reunion Island. The effects can be positive (rainfall favouring the creation of oviposition sites) or negative (heavy rainfall limiting the abundance of aquatic stages when cumulative rainfall over the preceding 35-day period is high) (Fig 3). This suggests that in Reunion Island, oviposition sites are mostly rainfall-dependent. This is in contrast with the situation in other areas, where *Ae*. *albopictus* females mainly breed in water containers that are independent of rainfall, for example those filled through the watering of plants in gardens [17, 48]. This result is consistent with the conclusions of observational studies conducted in different urban landscapes of Reunion Island, showing that oviposition sites can be either natural (*e*.*g*., tree holes) or artificial (*e*.*g*., water containers, flower plates or vases) [24]. The negative effect of rainfall on *Ae*. *albopictus* densities can be explained by the flushing of immature stages (eggs, larvae, pupae) from the rainfall-dependent oviposition sites under heavy precipitation [41]. Other climatic variables, such as relative humidity, were not included in our study although the impact of relative humidity on egg and adult stages has been reported [49]. In our study area, rainfall and temperature seem sufficient to explain most of the observed intra-annual variability in mosquito abundance, particularly because the relative humidity in Reunion Island’s tropical climate remains favourable to adult survival throughout the year. However, this variable may be of importance in other contexts.

### Contribution of the empirical modelling approach

A large set of explicative variables (531 variables) were tested in the exploratory empirical approach. Our results highlighted the importance of precipitation and temperature on the dynamics of *Ae*. *albopictus* larval stages in Reunion Island. They showed that the observed larvae abundance can be explained from the cumulative values of these two key variables over a time period of 5 to 8 weeks before the sampling with little differences between the first ten models in terms of performance (S2 Table). This time period is probably related to the reproductive biological life cycle of *Ae*. *albopictus*, as temperature impacts the development of immature stages [39], and rainfall has an effect on the availability of some of the oviposition sites. Our results, although based on the collection of immature stages collections alone, are consistent with other studies demonstrating the effect of cumulative temperature on host-seeking *Ae*. *albopictus* females [48]. Overall, the interpretation of the results of the empirical modelling approach helped to identify the mechanisms (*i*.*e*., the impact of rainfall on the oviposition sites) to be included in the process-based approach. The relationship between larval abundance and weather variables was found to be clearly non-linear (Fig 3). While the model predicts little or no abundance when mean temperatures and cumulative rainfall are low (respectively below 21°C and below 90 mm), the number of larvae can be observed when mean temperatures are low (< 21°C) and rainfall is heavy (last 35 days cumulative > 550 mm). The mean number of larval stages L3 and L4 per trap may exceed 40 when the average of minimum temperature over the last 42 days is above 22°C and the cumulative rainfall over the last 35 days is between 90 mm and 800 mm. Interestingly, even when the temperature conditions are *a priori* favourable (average above 22°C), if the cumulative rainfall is above 800 mm during the last 35 days, the predicted number of larvae does not exceed 20. The latter result may be linked to the flooding of oviposition sites, which may have a negative impact on the population dynamics of *Ae*. *albopictus* [41, 50]. This non-linear relationship cannot be handled by standard linear models and the SVM approach (among other empirical approaches that can handle non-linearity, such as generalized additive models) was particularly important in this context. Another interesting feature is that the best model calibrated with data from all sites displayed results comparable to models calibrated site by site (less than 3% of mean square error loss).

### Contribution of the process-based modelling approach

In this second approach, the biological processes underlying *Ae*. *albopictus* populations were described using a weather-driven mosquito population dynamics model [17] that was adapted to tropical *Ae*. *albopictus* populations present in Reunion Island. The impacts of rainfall and temperature on the development and mortality rates of aquatic and aerial stages were thus explicitly modelled based on a bibliographic review of observational and experimental studies. In this exercise, the results from the empirical approach were used in the identification of the main drivers of the population dynamics and the modelling choices. As outputs, the process-based model predicted the abundances of all the different stages. This allowed the validation of the model using entomological field data on larvae abundance (Fig 4), but also on egg collections (see S3 File), and predictions of the adult stages as an entomological risk index (Fig 5). In addition, such an approach, one that explicitly included the availability of oviposition sites for each operational zone used by the vector control service, rendered it possible to predict the mosquito abundances at an adequate spatial resolution for surveillance and control. Indeed, the model predictions are made for each operational zone defined by the vector control service to plan interventions.

### Complementarity of the two approaches

As far as we know, this is the first study implementing both empirical and process-based approaches to model mosquito population dynamics, although the relative advantages of both approaches have been well acknowledged in ecology [51, 52]. Previous studies have compared the respective potential of the two approaches to map the ecological niches of *Aedes albopictus* [53] and of pathogens it can transmit, such as Zika virus [54]. They highlight striking correspondence between the two approaches for modelling species distribution [52, 53], but discrepancies regarding forecasts under different climate change projections [53] or when coupling vector distribution with epidemiological dynamics [54]. Based on correlative relationships, empirical models are simpler, with the mechanisms implicit, but require data for the construction and the validation of the model. It should be noted that the range of values of the data used to build the model define the limits of model application. In our study, the five study sites used to build the empirical model are all located in coastal areas (Fig 1), thus the predictions of the empirical model in the mountainous interior of the island should be taken with caution. Moreover, the biological explanations of the empirical relationships may not be obvious, in the case of non-linear relationships and cumulative effects. Process-based models, on the other hand, are based on causal relationships, do not require data for their construction, and are more comprehensive because they explicitly incorporate mechanisms. Moreover, such models can be used to test *in silico* different scenarios, in particular, control strategies for aquatic and adult stages [19]. However, they require an important amount of knowledge of the biological processes involved that may not be available for species other than *Ae*. *albopictus*, for which numerous studies have been published in recent years. In our study, although enough knowledge on *Ae*. *albopictus* was available to build a process-based model, the conclusions of the empirical approach were useful as they allowed us to identify the main drivers of the population dynamics that were included in the process-based model. As all empirical models with the best performances included a set of variables related to rainfall and temperature (S2 Table), the two variables were identified as important. This result demonstrated that temperature remains an important driver of *Ae*. *albopictus* population dynamics in a tropical environment, although the annual temperature variations are less important than in a temperate climate. Thus, regarding this aspect the process-based model developed in Southern France [17] could not be simplified. Moreover, the empirical approach highlighted the negative effects of rainfall; this effect was explicitly taken into account in the process-based model, with the definition of rainfall-dependent mortality functions for aquatic stages. The comparison between modelled and observed abundances (Fig 4) showed that the two approaches can be successfully used to predict *Ae*. *albopictus* abundances over time from precipitation and temperature data. The comparison between the predictions of the two approaches demonstrated that the models are in strong agreement for climatic conditions similar to those of the study sites where entomological data were available to build the empirical model (S2 Fig). These conditions correspond to the coastal, more densely populated areas, where a good knowledge of the population dynamics of *Ae*. *albopictus* is needed for the organization of vector control measures by public health authorities. In these areas, the strong agreement of the two models strengthens the confidence in models’ predictions (S3 Fig). In other areas, the process-based approach should be preferred, as it is more reliable than the empirical approach to predict mosquito population dynamics in areas where the meteorological conditions differ from the observed dataset.

### Transfer of research results to stakeholders and decision-makers

The ‘ALBORUN’ tool developed in this study was successfully transferred to the vector control service of the Regional Health Agency as a mapping tool for the surveillance and control of vector populations. To the best of our knowledge, this tool is the first population dynamics model for *Ae*. *albopictus* populations in a tropical environment that simulates in time and space mosquito densities using daily meteorological data and environmental characteristics. It allows one to compare different areas and/or periods of time, and helps decision-makers target areas for surveillance and control. Whereas all recent modelling studies of *Ae*. *albopictus* distribution [6–8, 11, 12, 16] use environmental data such as the land cover or satellite-derived indices as proxies of the suitable areas for *Ae*. *albopictus*, the ‘ALBORUN’ tool relies on field data on the number of potential oviposition sites. From this large and exhaustive dataset (all operational zones are monitored by the Regional Health Agency services), the inputs of the ‘ALBORUN’ tool are as close to reality as possible, and indeed the model outputs reflect the high spatial heterogeneity of *Ae*. *albopictus* populations that is observed on Reunion Island [24].

### Limitations and perspectives

The empirical model with a set of two variables (cumulative rainfall over the last 35 days and the average of minimum temperature over the last 42 days) provided the best results in terms of Mean Square Error between observations and model outputs (S2 Table), using a 5-fold cross validation method by randomly assigning the data. Nevertheless, in the case of temporal data with temporal autocorrelation, assigning data non randomly in the cross-calibration process could be beneficial as soon as more data become available to avoid any risk of overfitting [55].

The main limitation of the process-based model is the uncertainty on the parameters and functions values (Tables 2 and 3). As they could not all be derived from studies on *Ae*. *albopictus* species in Reunion, we also had to turn to studies conducted in other geographical contexts. According to our expertise, the values are realistic for Reunion, but additional local experimental studies may improve the parameter and function estimates, and consequently the model outputs. In addition, it should be noted that the evaluation of the models was based on one-year entomological field data for four out of the five study sites.

New entomological collections in varied eco-climatic zones of Reunion Island would be needed to assess the validity of the ‘ALBORUN’ tool at a regional scale. In particular, standard mark-release-recapture experiments [56] would allow one to evaluate the capacity of the process-based model to predict the absolute values of mosquito densities. Moreover, such experiments would allow evaluating the impact of mosquito dispersal on the population dynamics. In this study, this impact was neglected, assuming the inflows/outflows of mosquitoes negligible compared to the population of a zone, as Lacroix et al. (2009) demonstrated that in Reunion Island *Ae*. *albopictus* has a short dispersal range (less than 50 m), and as the operational zones were defined by delineating isolated areas, or areas separated by barriers for mosquito dispersal such as roads and open fields [31] (S1 Fig). Yet, such assumption would need to be verified from field observations.

Finally, in our study we compared two modelling approaches with the only and limited example of *Ae*. *albopictus* in Reunion Island. Assessing the respective performances of the two approaches for other geographical contexts and/or species would allow a broader and less specific comparison.

Due to the particular relief and climate of Reunion Island, rainfall and temperature data can vary greatly over short distances. Finer estimates of local rainfall and temperature (*e*.*g*. gridded rainfall and temperature data) would improve the model predictions. Other potential improvements of the tool include the possibility to integrate and test control actions [19], either standard operations such as the mechanical destruction of oviposition sites or insecticide spraying, or techniques that could be developed in future years such as the Sterile Insect Technique (SIT) or autodissemination [25, 57]. This would make it possible to test the effects of different control strategies in a realistic environment, with operational recommendations for the implementation of integrated control. Such improvements imply transforming the process-based model into a stochastic model in order to simulate possible population extinction, and accounting for mosquito dispersal between neighbouring zones [26]. Moreover, the model of mosquito population dynamics could be combined with an epidemiological model of transmission to predict the areas at risk of transmission for different pathogens, such as dengue, chikungunya, or Zika viruses. Another perspective would be to apply the ‘ALBORUN’ model in other tropical areas and test its genericity. Due to the way it was constructed, the process-based model of *Ae*. *albopictus* developed for Reunion Island can be used in other areas, with a geographic breakdown adapted to the needs of health and vector control policy makers. However, its application may be limited by the availability of environmental input data. Indeed, in Reunion the network of weather stations is very dense (Fig 1), and accurate data on the spatial distribution of oviposition sites are collected on a regular basis by the vector control service of the Regional Health Agency. In data-scarce contexts, satellite remote sensing techniques can provide alternative sources of such environmental data, either from high or very high spatial resolution imagery to assess the availability of oviposition sites [58], or imagery with a frequent revisit period to derive rainfall or temperature proxies [59]. The implementation of remote sensing-based population dynamics models would represent a significant advance in the development and dissemination of operational tools for real-time monitoring of vector-borne diseases.

## Supporting information

S1 Fig Examples of operational zones defined by Regional Health Agency, Reunion Island. (TIF)

S2 Fig Plot showing the effect of temperature and rainfall on the agreement between the predictions of empirical and process-based models. (TIFF)

S3 Fig Map of the agreement between the predictions of empirical and process-based models, Reunion Island. (TIF)

S1 Table Results of *Aedes albopictus* larvae collections, Reunion Island, 2012–2013. (PDF)

S2 Table List of the ten empirical models with the best performances in terms of mean square error with a 5-fold cross validation method. (PDF)

S1 File *Aedes albopictus* egg collections used to assess the process-based population dynamics model. (PDF)

S2 File Estimation of the environment carrying capacities of *Aedes albopictus* aquatic stages in Reunion Island. (PDF)

S3 File Assessment of the process-based population dynamics model–comparison with *Aedes albopictus* egg collections. (PDF)

S4 File Presentation of ‘ALBORUN’ tool. (PDF)
