# Supplementary figures and images for: Complementarity of empirical and process-based approaches to modelling mosquito population dynamics with Aedes albopictus as an example—Application to the development of an operational mapping tool of vector populations
Source: PLoS One. 2020 Jan 17;15(1):e0227407. doi: 10.1371/journal.pone.0227407 (PMC6968851; doi:10.1371/journal.pone.0227407)

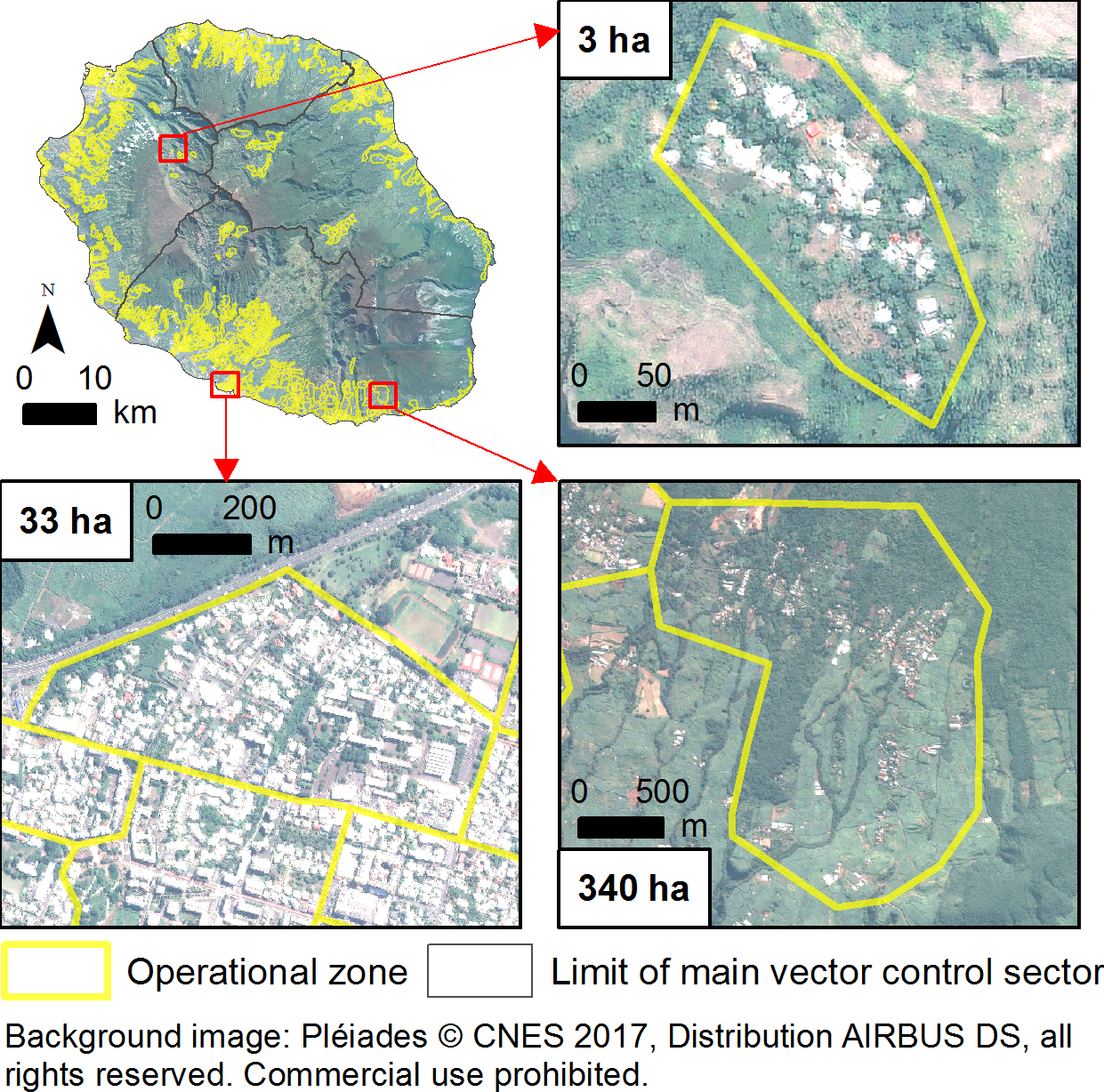

Supplement: S1 Fig — (TIF) [file pone.0227407.s001.tif]

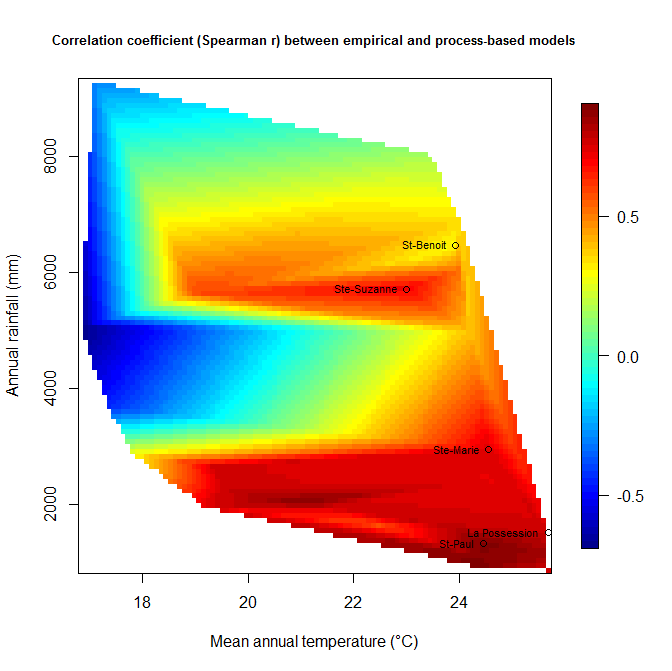

Supplement: S2 Fig — (TIFF) [file pone.0227407.s002.tiff]

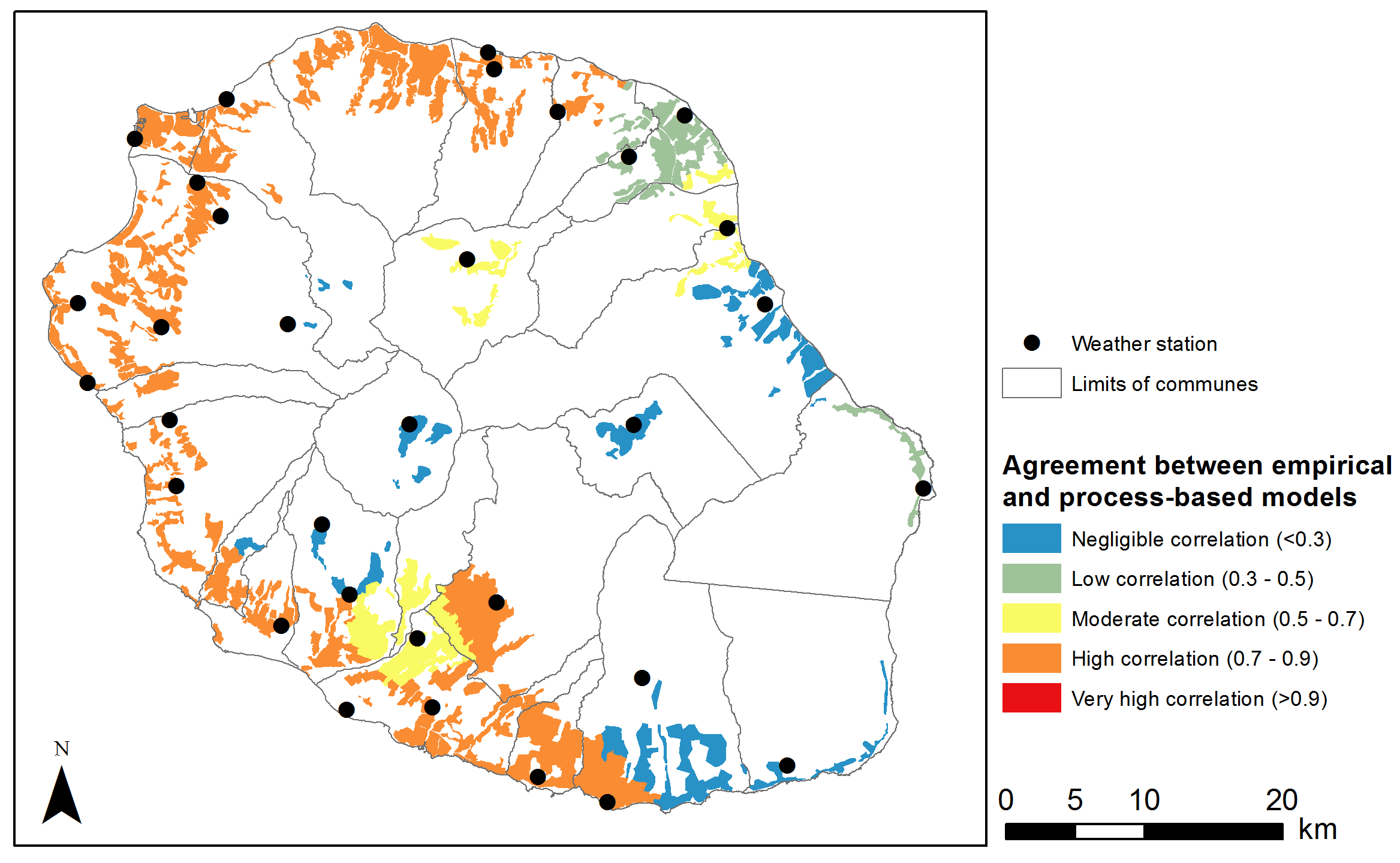

Supplement: S3 Fig — (TIF) [file pone.0227407.s003.tif]
