# Supplementary material for: Complementarity of empirical and process-based approaches to modelling mosquito population dynamics with Aedes albopictus as an example—Application to the development of an operational mapping tool of vector populations
Source: PLoS One. 2020 Jan 17;15(1):e0227407. doi: 10.1371/journal.pone.0227407 (PMC6968851; doi:10.1371/journal.pone.0227407)
Supplement: S1 Table — (PDF) [file pone.0227407.s004.pdf]

Supplementary Information for

Complementarity of empirical and process-based approaches to modelling mosquito population dynamics with *Aedes albopictus* as an example – application to the development of an operational mapping tool of vector populations

Annelise Tran, Morgan Mangeas, Marie Demarchi, Emmanuel Roux, Pascal Degenne, Marion Haramboure, Gilbert Le Goff, David Damiens, Louis-Clément Gouagna, Vincent Herbreteau, Jean-Sébastien Dehecq

Corresponding author: Annelise Tran
Email: annelise.tran@cirad.fr

S1 Table: Results of *Aedes albopictus* larvae collections, Reunion Island, 2012-2013.

Site	Date	Mean larvae per trap
La Possession	31/10/2012	0.0
La Possession	06/11/2012	0.0
La Possession	14/11/2012	0.0
La Possession	20/11/2012	0.2
La Possession	28/11/2012	2.4
La Possession	05/12/2012	22.8
La Possession	12/12/2012	15.2
La Possession	18/12/2012	16.0
La Possession	26/12/2012	26.0
La Possession	02/01/2013	47.4
La Possession	08/01/2013	34.0
La Possession	16/01/2013	24.0
La Possession	23/01/2013	16.6
La Possession	30/01/2013	16.8
La Possession	06/02/2013	31.8
La Possession	14/02/2013	26.0
La Possession	20/02/2013	23.4
La Possession	28/02/2013	47.8
La Possession	06/03/2013	37.2
La Possession	14/03/2013	45.2
La Possession	19/03/2013	38.4
La Possession	28/03/2013	38.8

Site	Date	Mean larvae per trap
St-Benoit	16/02/2012	0.0
St-Benoit	22/02/2012	8.0
St-Benoit	29/02/2012	16.0
St-Benoit	07/03/2012	12.5
St-Benoit	14/03/2012	16.3
St-Benoit	21/03/2012	3.0
St-Benoit	28/03/2012	3.7
St-Benoit	04/04/2012	42.3
St-Benoit	11/04/2012	15.2
St-Benoit	18/04/2012	9.2
St-Benoit	25/04/2012	13.0
St-Benoit	02/05/2012	7.3
St-Benoit	09/05/2012	18.8
St-Benoit	15/05/2012	9.0
St-Benoit	23/05/2012	19.2
St-Benoit	30/05/2012	15.0
St-Benoit	06/06/2012	10.3

Site	Date	Mean larvae per trap
Ste-Marie	16/02/2012	0.0
Ste-Marie	22/02/2012	15.3
Ste-Marie	29/02/2012	29.5
Ste-Marie	07/03/2012	65.5
Ste-Marie	14/03/2012	53.3
Ste-Marie	21/03/2012	75.0
Ste-Marie	28/03/2012	24.7
Ste-Marie	04/04/2012	48.3
Ste-Marie	11/04/2012	54.8
Ste-Marie	18/04/2012	15.7
Ste-Marie	25/04/2012	10.3
Ste-Marie	02/05/2012	46.0
Ste-Marie	09/05/2012	50.2
Ste-Marie	15/05/2012	25.3
Ste-Marie	23/05/2012	18.7
Ste-Marie	30/05/2012	9.0
Ste-Marie	06/06/2012	1.7
Ste-Marie	31/10/2012	0.0
Ste-Marie	06/11/2012	0.0
Ste-Marie	14/11/2012	27.0
Ste-Marie	20/11/2012	4.6
Ste-Marie	28/11/2012	16.6
Ste-Marie	05/12/2012	14.4
Ste-Marie	12/12/2012	12.2
Ste-Marie	18/12/2012	4.4
Ste-Marie	26/12/2012	32.6
Ste-Marie	02/01/2013	10.6
Ste-Marie	08/01/2013	47.8
Ste-Marie	16/01/2013	10.6
Ste-Marie	23/01/2013	36.8
Ste-Marie	30/01/2013	75.4
Ste-Marie	06/02/2013	71.6
Ste-Marie	14/02/2013	35.6
Ste-Marie	20/02/2013	38.4
Ste-Marie	28/02/2013	35.2
Ste-Marie	06/03/2013	73.2
Ste-Marie	14/03/2013	76.8
Ste-Marie	19/03/2013	20.6
Ste-Marie	28/03/2013	63.6

Site	Date	Mean larvae per trap
Ste-Suzanne	31/10/2012	0.0
Ste-Suzanne	06/11/2012	11.6
Ste-Suzanne	14/11/2012	23.6
Ste-Suzanne	20/11/2012	8.8
Ste-Suzanne	28/11/2012	12.8
Ste-Suzanne	05/12/2012	10.6
Ste-Suzanne	12/12/2012	5.6
Ste-Suzanne	18/12/2012	4.8
Ste-Suzanne	26/12/2012	13.0
Ste-Suzanne	02/01/2013	7.4
Ste-Suzanne	08/01/2013	17.0
Ste-Suzanne	16/01/2013	20.0
Ste-Suzanne	23/01/2013	22.4
Ste-Suzanne	30/01/2013	14.0
Ste-Suzanne	06/02/2013	15.8
Ste-Suzanne	14/02/2013	15.4
Ste-Suzanne	20/02/2013	11.0
Ste-Suzanne	28/02/2013	20.8
Ste-Suzanne	06/03/2013	19.6
Ste-Suzanne	14/03/2013	27.2
Ste-Suzanne	19/03/2013	6.2
Ste-Suzanne	28/03/2013	14.2

Site	Date	Mean larvae per trap
St-Paul	31/10/2012	0.0
St-Paul	06/11/2012	0.0
St-Paul	14/11/2012	0.0
St-Paul	20/11/2012	12.0
St-Paul	28/11/2012	0.0
St-Paul	05/12/2012	1.6
St-Paul	12/12/2012	0.0
St-Paul	18/12/2012	1.0
St-Paul	26/12/2012	18.2
St-Paul	02/01/2013	26.0
St-Paul	08/01/2013	18.8
St-Paul	16/01/2013	38.4
St-Paul	23/01/2013	30.6
St-Paul	30/01/2013	36.0
St-Paul	06/02/2013	56.2
St-Paul	14/02/2013	26.8
St-Paul	20/02/2013	33.4
St-Paul	28/02/2013	60.4
St-Paul	06/03/2013	42.0
St-Paul	14/03/2013	26.4
St-Paul	19/03/2013	4.0
St-Paul	28/03/2013	29.8
