# Supplementary material for: Complementarity of empirical and process-based approaches to modelling mosquito population dynamics with Aedes albopictus as an example—Application to the development of an operational mapping tool of vector populations
Source: PLoS One. 2020 Jan 17;15(1):e0227407. doi: 10.1371/journal.pone.0227407 (PMC6968851; doi:10.1371/journal.pone.0227407)
Supplement: S2 Table — (PDF) [file pone.0227407.s005.pdf]

Supplementary Information for

Complementarity of empirical and process-based approaches to modelling mosquito population dynamics with *Aedes albopictus* as an example – application to the development of an operational mapping tool of vector populations

Annelise Tran, Morgan Mangeas, Marie Demarchi, Emmanuel Roux, Pascal Degenne, Marion Haramboure, Gilbert Le Goff, David Damiens, Louis-Clément Gouagna, Vincent Herbreteau, Jean-Sébastien Dehecq

Corresponding author: Annelise Tran
Email: annelise.tran@cirad.fr

S2 Table: List of the ten models with the best performances in terms of mean square error with a 5-fold cross validation method. Models are ordered from best to worst. *TN_mean_N* denotes the average minimum temperature over the last *N* days, *RR_log_cumul_N* denotes the logarithm of cumulative rainfall over the last *N* days and TN_nb_d_*N*_inf_*S* denotes the number of days during the last N days for which the average temperature was less than *S* °C.

Input 1	Input 2	Input 3	Mean Square Error
RR_log_cumul_35	TN_mean_42		240.25
RR_log_cumul_35	TN_nb_d_35_inf_21.7		240.8
RR_log_cumul_35	TN_mean_35	TN_nb_d_42_inf_22	243.15
RR_log_cumul_49	TN_mean_56		246.99
RR_log_cumul_49	TN_mean_49	TN_nb_d_56_inf_22	247.03
RR_log_cumul_35	TN_nb_d_35_inf_22		248.62
RR_log_cumul_35	TN_nb_d_56_inf_21.7		249.25
RR_log_cumul_56	TN_mean_56	TN_mean_49	251.35
RR_log_cumul_49	TN_nb_d_56_inf_21.7	TN_mean_49	251.36
RR_log_cumul_56	TN_nb_d_35_inf_22		252.20
