# Supplementary material for: Complementarity of empirical and process-based approaches to modelling mosquito population dynamics with Aedes albopictus as an example—Application to the development of an operational mapping tool of vector populations
Source: PLoS One. 2020 Jan 17;15(1):e0227407. doi: 10.1371/journal.pone.0227407 (PMC6968851; doi:10.1371/journal.pone.0227407)
Supplement: S1 File — (PDF) [file pone.0227407.s006.pdf]

Supplementary Information for

Complementarity of empirical and process-based approaches to modelling mosquito population dynamics with *Aedes albopictus* as an example – application to the development of an operational mapping tool of vector populations

Annelise Tran, Morgan Mangeas, Marie Demarchi, Emmanuel Roux, Pascal Degenne, Marion Haramboure, Gilbert Le Goff, David Damiens, Louis-Clément Gouagna, Vincent Herbreteau, Jean-Sébastien Dehecq

S1 File: *Aedes albopictus* egg collections used to assess the process-based population dynamics model

For the comparison with the process-based model outputs, the Duparc site (2 operational zones of 14 and 16 ha) located close to the Sainte-Marie ‘La Mare’ weather station was selected. Egg collections were performed weekly between May 2013 and August 2014, using ovitraps in different sites in Sainte-Marie (Latitude: 20° 52' 60"S. Longitude: 55° 33' 58’’ E) (Figure S1a, Table S1).

A total of 24 ovitraps, which are artificial egg-laying containers, were randomly deployed in the Duparc study site, and were set up in private human dwellings or public areas, usually at ground level in shaded locations. Each ovitrap consists of a 1 liter black plastic container (13 cm in diameter x 12 cm in height) that is partially filled with 650 ml of river water or tap water. The oviposition substrate was made of a brown germination paper clipped on a 15 x 10 cm stiff PVC sheet.

Taking into account the weekly frequency of the ovitrap survey, the presence of larvae, especially pupae and / or pupal exuvia, was regularly monitored to prevent emergence. Oviposition activity and the number of eggs laid were monitored weekly. Upon removal, the substrates were left to dry under room temperature for at least 24 hours before the number of eggs from each of substrate was recorded under a dissecting microscope. Subsequently, egg papers were air dried for 5 days in a laboratory and dipped into a solution containing diluted dehydrated rabbit food suspension to induce hatching. The hatching rate was determined by counting the number of hatched and unhatched eggs under a stereomicroscope.

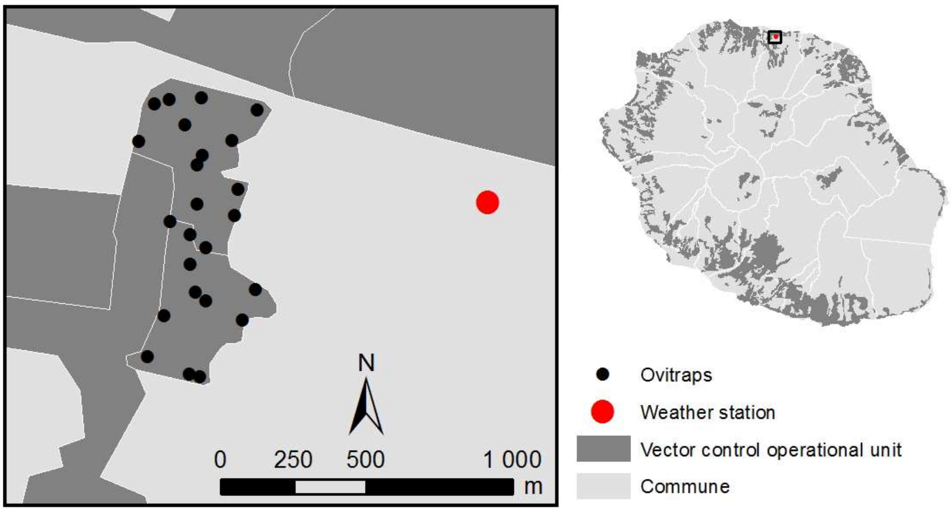

Figure S1a: Location of the ovitraps in the Duparc study site.

Table S1. Results of egg collections, Duparc site, 2013-2015

Collection date (DD/MM/YYYY)	Eggs / trap
21/05/2013	124.7
28/05/2013	119.1
04/06/2013	110.2
11/06/2013	79.6
18/06/2013	63.3
25/06/2013	47.4
02/07/2013	41.5
09/07/2013	58.6
16/07/2013	37.9
23/07/2013	30.0
31/07/2013	24.9
01/08/2013	125.0
06/08/2013	17.7
13/08/2013	27.6
14/08/2013	14.0
20/08/2013	30.0
27/08/2013	27.4
03/09/2013	28.0
10/09/2013	30.7
17/09/2013	51.9
24/09/2013	49.0
01/10/2013	48.8
08/10/2013	28.9
15/10/2013	37.5
22/10/2013	29.0
29/10/2013	31.5
05/11/2013	35.8
12/11/2013	36.4
19/11/2013	63.3
26/11/2013	55.7
03/12/2013	56.9
09/12/2013	121.0
17/12/2013	157.1

Collection date (DD/MM/YYYY)	Eggs / trap
14/01/2014	80.6
21/01/2014	134.8
28/01/2014	103.0
04/02/2014	98.3
11/02/2014	118.1
18/02/2014	79.9
25/02/2014	68.0
04/03/2014	65.7
11/03/2014	56.6
18/03/2014	42.2
25/03/2014	41.7
01/04/2014	30.4
08/04/2014	37.6
15/04/2014	65.6
22/04/2014	54.3
29/04/2014	62.9
06/05/2014	48.3
13/05/2014	51.4
20/05/2014	44.1
27/05/2014	47.8
03/06/2014	71.7
10/06/2014	37.7
17/06/2014	49.1
24/06/2014	53.2
01/07/2014	25.3
08/07/2014	38.7
15/07/2014	27.9
22/07/2014	36.0
29/07/2014	38.4
05/08/2014	24.5
12/08/2014	35.1
19/08/2014	39.5
26/08/2014	18.6

Collection date (DD/MM/YYYY)	Eggs / trap
23/06/2015	115.9
30/06/2015	144.3
07/07/2015	225.2
13/07/2015	117.7
21/07/2015	133.9
28/07/2015	95.4
04/08/2015	109.9
11/08/2015	152.7
18/08/2015	76.2
24/08/2015	140.9
01/09/2015	125.3
08/09/2015	64.2
15/09/2015	59.6
22/09/2015	65.6
29/09/2015	56.3
06/10/2015	93.2
13/10/2015	96.8
20/10/2015	63.0
27/10/2015	66.9
03/11/2015	80.3
10/11/2015	116.0
