# Supplementary material for: Complementarity of empirical and process-based approaches to modelling mosquito population dynamics with Aedes albopictus as an example—Application to the development of an operational mapping tool of vector populations
Source: PLoS One. 2020 Jan 17;15(1):e0227407. doi: 10.1371/journal.pone.0227407 (PMC6968851; doi:10.1371/journal.pone.0227407)

Supplementary Information for

Complementarity of empirical and process-based approaches to modelling mosquito population dynamics with *Aedes albopictus* as an example – application to the development of an operational mapping tool of vector populations

Annelise Tran, Morgan Mangeas, Marie Demarchi, Emmanuel Roux, Pascal Degenne, Marion Haramboure, Gilbert Le Goff, David Damiens, Louis-Clément Gouagna, Vincent Herbreteau, Jean-Sébastien Dehecq

Corresponding author: Annelise Tran
Email: annelise.tran@cirad.fr

S2 File: Estimation of the environment carrying capacities of *Aedes albopictus* aquatic stages in Reunion Island

***Ae. albopictus* monitoring data.** During inter-epidemic periods, vector control in Reunion Island consists in the surveillance of the operational zones defined by the Regional Health Agency. In average over the 2007-2015 period, 9 zones were visited per day, and within these zones, 77 houses were inspected. Thus, each of the 1,203 operation zone is visited at least once a year. In each house, the outdoor natural and artificial potential breeding sites are inspected and then systematically destroyed. For each visit and each operational zone, the number of visited houses, the number of potential mosquitoes' breeding sites and their types are recorded. The typology of *Ae. albopictus* breeding sites includes the following types: flower pots and vases, flower plates, small containers (<10 l), large containers (>10 l), natural breeding sites, used tires, ponds and pools, others. Between 2007 and 2008, additional surveys were conducted in the different communes to evaluate the number of L3/L4 larvae per breeding site according to their typology (Table S2).

Table S2. Detailed typology and productivity of *Aedes albopictus* breeding sites in Reunion Island

Filling	Breeding site	Mean number of L3/L4 larvae [min-max]
Rainfall-independent	Flower pots and vases	124 [2 – 583]
	Flower plates	22 [0 – 196]
Rainfall-dependent	Small containers (<10 l)	57 [0 – 368]
	Large containers (>10 l)	53 [0 – 366]
	Natural breeding sites	15 [0 – 154]
	Used tires	93 [1 – 923]
	Ponds and pools	98 [4 – 549]

Estimation of the environment carrying capacities of *Ae. albopictus.* The calculation, for each operational zone, of the environment carrying capacities of *Ae. albopictus* aquatic stages includes different steps.

First, for each operational zone *z* and each date *t*, the total number of potential breeding sites of type *i* (n_i) was estimated by multiplying the observed number of potential breeding sites of type *i* by the proportion of visited houses:

$$n_i(z,t) = n_{iobs}(z,t) * \frac{H_{visit}(z,t)}{Htot(z)} \qquad \text{(Eq. S2.1)}$$

where $H_{visit}(z,t)$ is the number of visited houses in the operational zone *z* at time *t*, $H_{tot}(z)$ the total number of houses in zone *z*, *i* in {flower pots and vases, flower plates, small containers (<10 l), large containers (>10 l), natural breeding sites, used tires, ponds and pools}.

Second, the average number of potential breeding sites observed per operational zone and per type was computed over the period 2010-2015 (Figure S2a). This period of time was chosen to be representative of the period when the entomological collections took place (2012-2014).

Finally, the fixed and variable environment carrying capacities of *Ae. albopictus* larvae (κ_{Lfix} and κ_{Lvar}) were estimated as follows (Figure S2b):

$$\kappa_{Lfix}(z) = \sum_i n_i\,(z).\,l_i \qquad \text{(Eq. S2.2)}$$

where $n_i(z)$ is the average number of potential breeding sites of type *i* in the operational zone z, and l_i the average number of L3/L4 larvae observed / breeding site of type *i* (Table S2), *i* in {flower pots and vases, flower plates}.

$$\kappa_{Lvar}(z) = \sum_i n_i\,(z).\,l_i \qquad \text{(Eq. S2.3)}$$

where $n_i(z)$ is the average number of potential breeding sites of type *i* in the operational zone z, and l_i the average number of L3/L4 larvae observed / breeding site of type *i* (Table S2), *i* in {small containers (<10 l), large containers (>10 l), natural breeding sites, used tires, ponds and pools}.

The fixed and variable environment carrying capacities of *Ae. albopictus* pupae (κ_{Pfix} and κ_{Pvar}) were estimated to be equal to the fixed and variable environmental carrying capacities of larvae.

Figure S2a. ***Aedes albopictus*** **potential breeding sites densities, Reunion Island.**

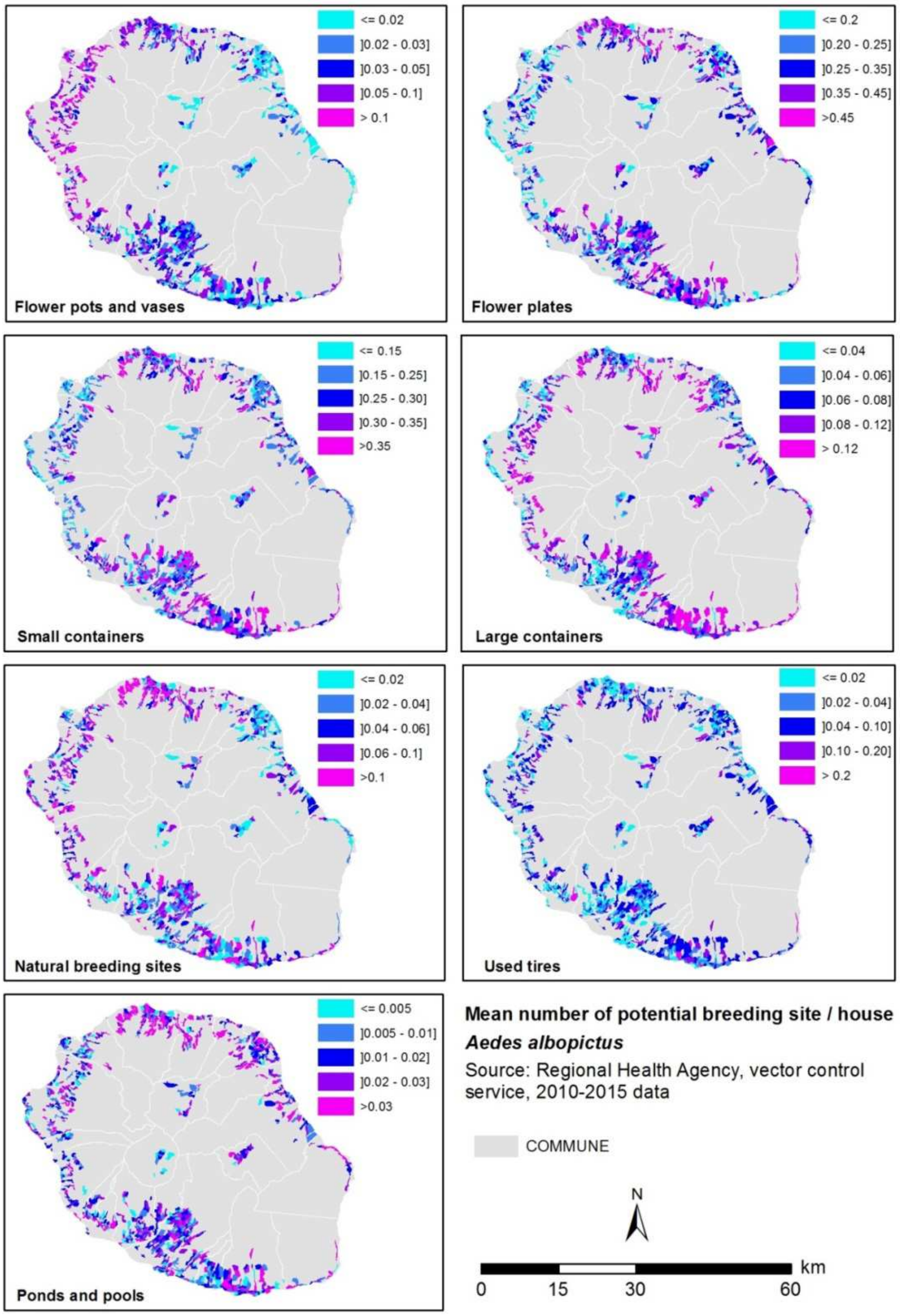

Figure S2b. Estimated fixed and variable environment carrying capacities of *Aedes albopictus* aquatic stages, Reunion Island.

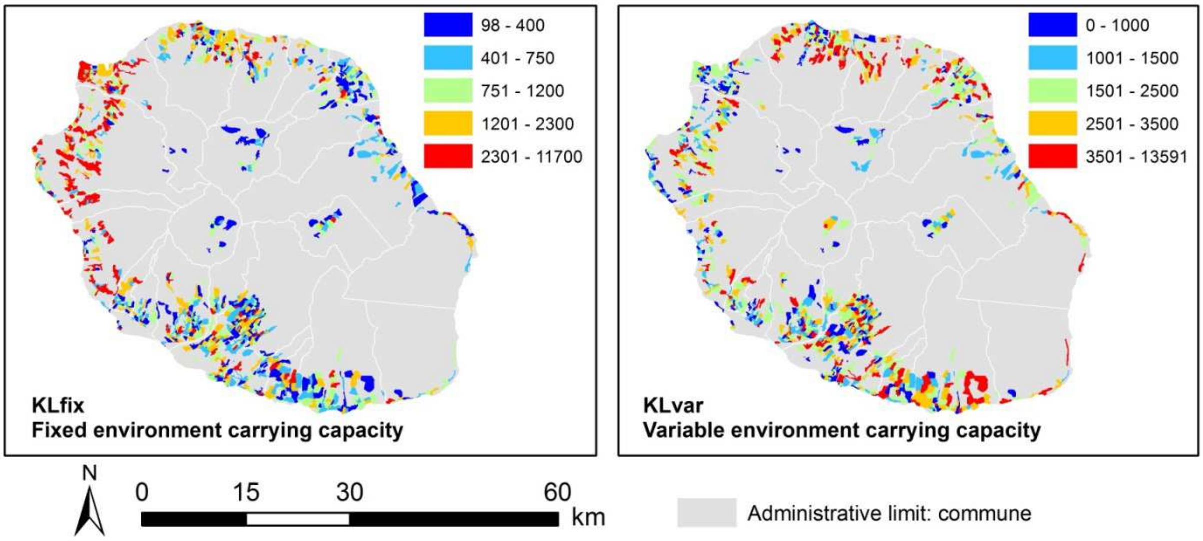

Supplement: S2 File — (PDF) [file pone.0227407.s007.pdf]
