# Supplementary material for: Complementarity of empirical and process-based approaches to modelling mosquito population dynamics with Aedes albopictus as an example—Application to the development of an operational mapping tool of vector populations
Source: PLoS One. 2020 Jan 17;15(1):e0227407. doi: 10.1371/journal.pone.0227407 (PMC6968851; doi:10.1371/journal.pone.0227407)
Supplement: S3 File — (PDF) [file pone.0227407.s008.pdf]

# Supplementary Information for

**Complementarity of empirical and process-based approaches to modelling mosquito population dynamics with *Aedes albopictus* as an example – application to the development of an operational mapping tool of vector populations**

Annelise Tran, Morgan Mangeas, Marie Demarchi, Emmanuel Roux, Pascal Degenne, Marion Haramboure, Gilbert Le Goff, David Damiens, Louis-Clément Gouagna, Vincent Herbreteau, Jean-Sébastien Dehecq

Corresponding author: Annelise Tran
Email: annelise.tran@cirad.fr

**S3 file: Assessment of the process-based population dynamics model – comparison with *Aedes albopictus* egg collections.**

**Objective.** The objective of the comparison of the outputs of the process-based model with egg collections was to assess the capacity of the process-based model to depict the seasonal egg population dynamics.

**Entomological data.** Egg collections were used for the validation of the process-based model. They are described in S1 File.

**Meteorological data.** We used the daily temperature (minimum and maximum) and rainfall recorded from 2012 to 2014 by the closest weather service station as input of the process-based model.

**Simulation.** The model implemented in Ocelet (See Material and Methods section) was used to simulate *Ae. albopictus* population abundances over 2 consecutive years (2013/01/01-2014/12/31) after a starting period of one year. Initially, the population consisted only of eggs ($E(t=t0)=10^6$, with t0=2012/01/01).

**Evaluation of the process-based model.** We compared the observed average number of eggs per trap with the simulated abundances of eggs (*E*). The degree of association between observed and simulated number of eggs at the time of ovitrap collection was by calculating the Spearman correlation coefficient. The predicted egg abundance was consistent with the observed *Ae. albopictus* egg abundance (Figure S3). Predicted and observed egg dynamics were significantly correlated (Spearman r correlation coefficient r=0.68, $p<10^{-5}$).

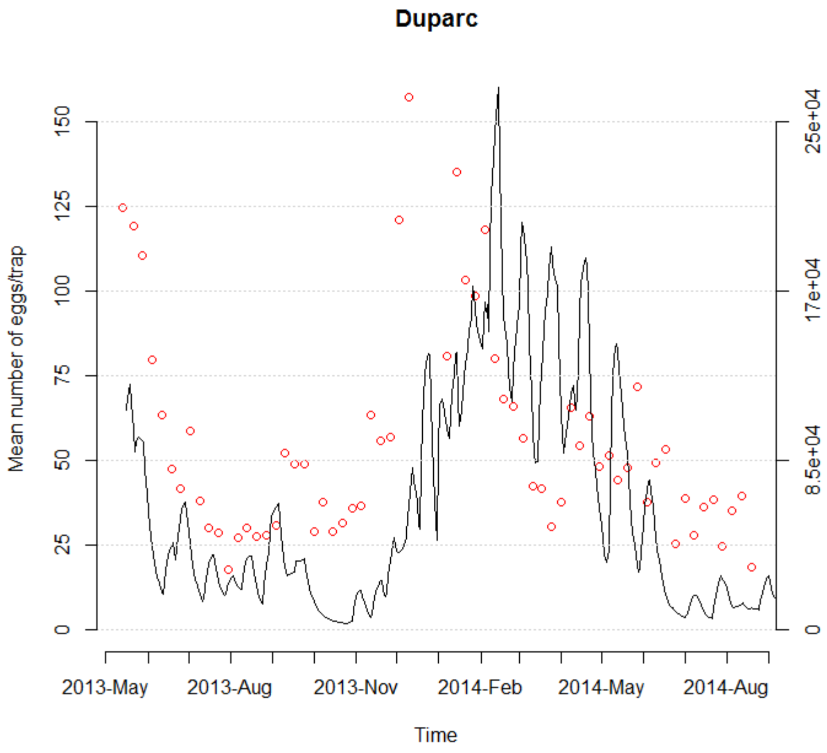


**Figure S3: Comparison of observed (red dots) and predicted (black line) abundances in *Aedes albopictus* eggs from rainfall and temperature data, Duparc study site, Reunion Island, 2013-2014.**
