# Supplementary material for: Complementarity of empirical and process-based approaches to modelling mosquito population dynamics with Aedes albopictus as an example—Application to the development of an operational mapping tool of vector populations
Source: PLoS One. 2020 Jan 17;15(1):e0227407. doi: 10.1371/journal.pone.0227407 (PMC6968851; doi:10.1371/journal.pone.0227407)
Supplement: S4 File — (PDF) [file pone.0227407.s009.pdf]

Supplementary Information for

Complementarity of empirical and process-based approaches to modelling mosquito population dynamics with *Aedes albopictus* as an example – application to the development of an operational mapping tool of vector populations

Annelise Tran, Morgan Mangeas, Marie Demarchi, Emmanuel Roux, Pascal Degenne, Marion Haramboure, Gilbert Le Goff, David Damiens, Louis-Clément Gouagna, Vincent Herbreteau, Jean-Sébastien Dehecq

Corresponding author: Annelise Tran
Email: annelise.tran@cirad.fr

S4 File: Presentation of 'ALBORUN' tool

The 'ALBORUN' tool is a user-friendly interface used to run the predictive models of *Aedes albopictus* populations in Reunion Island. The inputs of the tool are the *i)* limits of the operational zones used by the vector control service of the Regional Health Agency; *ii)* locations of the weather stations; and *iii)* meteorological data (daily temperatures and rainfall) provided for each station in a table format (Figure S4a).

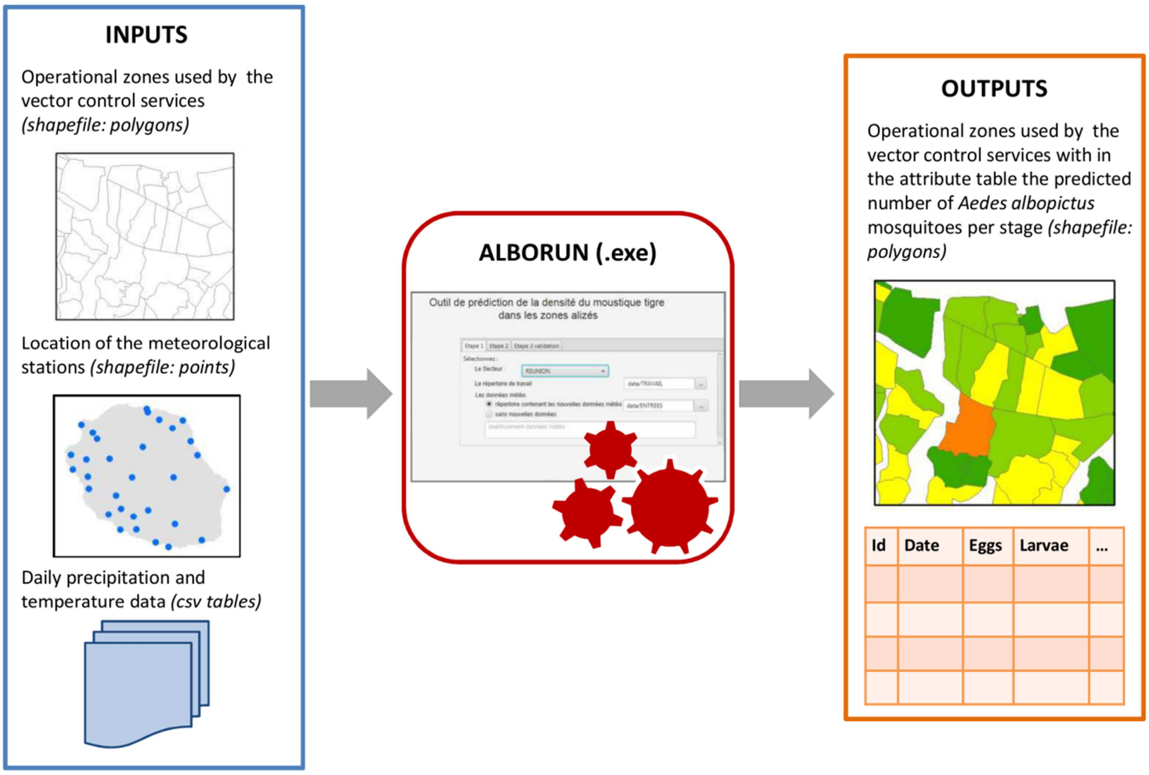

Figure S4a: Schematic presentation of the inputs and outputs of the ALBORUN tool

In the user interface, two tabs are available for setting the properties of the simulation (sector, start and end dates, output type, input and output folders).

On the third tab, a summary of the user's choices is displayed, and the user can run the simulation (Figure S4b).

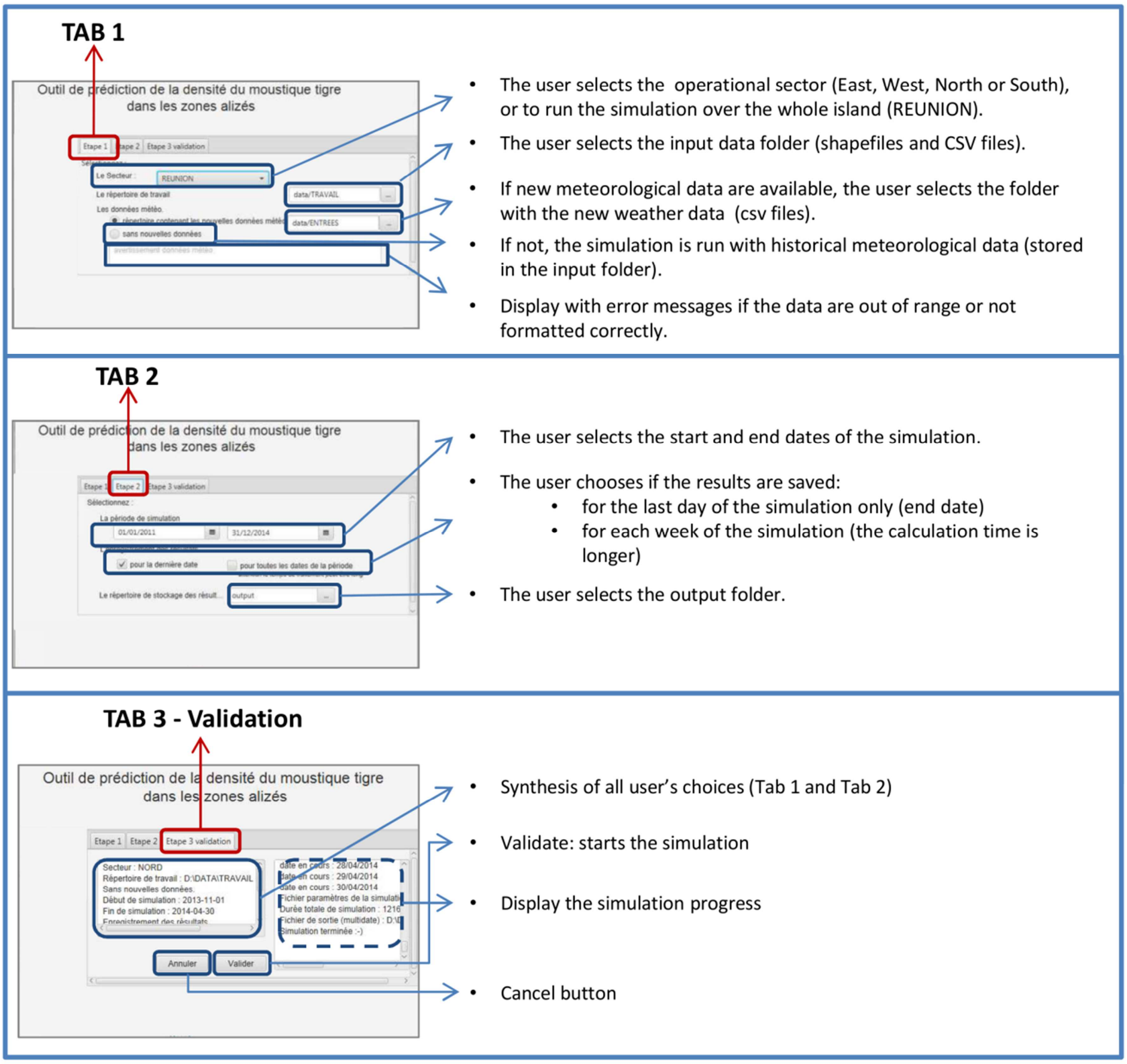

Figure S4b: ‘ALBORUN’ user interface.
